# 3D-Printed Carbon-Based Electrochemical Energy Storage Devices: Material Design, Structural Engineering, and Application Frontiers

**DOI:** 10.3390/ma18225070

**Published:** 2025-11-07

**Authors:** Yu Dong, Li Sun, Jiemin Dong, Wenhao Zou, Wan Rong, Jianfei Liu, Hanqi Meng, Qigao Cao

**Affiliations:** 1 State Key Laboratory of Porous Metal Materials, Northwest Institute for Nonferrous Metal Research, Xi’an 710016, China; dy960124@mail.ustc.edu.cn (Y.D.);; 2 Department of Applied Chemistry, School of Chemistry and Materials Science, University of Science and Technology of China, Hefei 230026, China

**Keywords:** 3D printing, carbon-based materials, energy storage

## Abstract

With the global energy structure transitioning towards clean and low-carbon alternatives, electrochemical energy storage technologies have emerged as pivotal enablers for achieving efficient renewable energy utilization and carbon neutrality objectives. However, conventional electrode materials remain constrained by inherent limitations, including low specific surface area, sluggish ion diffusion kinetics, and insufficient mechanical stability, which fundamentally hinder the synergistic fulfillment of high energy density, superior power density, and prolonged cycling durability. Three-dimensional printing technology offers a revolutionary paradigm for designing and fabricating carbon-based electrochemical energy storage devices. By enabling precise control over both the microstructural architecture and macro-scale morphology of electrode materials, this additive manufacturing approach significantly enhances energy/power densities, as well as cycling stability. Specifically, 3D printing facilitates biomimetic topological designs (e.g., hierarchical porous networks, vertically aligned ion channels) and functional hybridization strategies (e.g., carbon/metal oxide hybrids, carbon/biomass-derived composites), thereby achieving synergistic optimization of charge transfer kinetics and mechanical endurance. This review systematically summarizes recent advancements in 3D-printed carbon-based electrodes across major energy storage systems, including supercapacitors, lithium-ion batteries, and metal–air batteries. Particular emphasis is placed on the design principles of carbon-based inks, multiscale structural engineering strategies, and process optimization methodologies. Furthermore, we prospect future research directions focusing on smart 4D printing-enabled dynamic regulation, multi-material integrated systems, and artificial intelligence-guided design frameworks to bridge the gap between laboratory prototypes and industrial-scale applications. Through multidisciplinary convergence spanning materials science, advanced manufacturing, and device engineering, 3D-printed carbon electrodes are poised to catalyze the development of next-generation high-performance, customizable energy storage systems.

## 1. Introduction

### 1.1. The Demand and Challenges of the Era of Electrochemical Energy Storage

Human society is undergoing a profound transition from fossil fuels to sustainable and renewable energy sources. To achieve the ambitious goals of “carbon peak” and “carbon neutrality,” the large-scale grid integration of intermittent renewable energy sources such as wind and solar power, along with the rapid development of electric vehicles, smart grids, and portable electronic devices, has created an unprecedented demand for efficient, reliable, and safe electrochemical energy storage systems [1,2]. Electrochemical energy storage devices, such as lithium-ion batteries and supercapacitors, serve as the core medium for energy storage and conversion. Their performance directly determines the efficiency, lifespan, and cost of the entire energy system [3].

The electrode is the “heart” of an electrochemical energy storage device, and its performance fundamentally depends on two core elements: the intrinsic properties of the active materials and the multiscale architecture of the electrode [4]. Conventional electrode manufacturing processes (e.g., blade coating, roller pressing) typically involve coating a slurry of active materials, conductive agents, and binders onto two-dimensional planar current collectors. Although these processes are mature and easily scalable, their inherent limitations are increasingly becoming critical barriers to further performance improvements. Firstly, the fabricated electrodes often exhibit a monolithic, dense, and disordered thick-layer morphology, resulting in tortuous and lengthy ion transport pathways. Under conditions of high areal loading and high-rate charging/discharging, sluggish ion diffusion kinetics lead to significant polarization and capacity decay [5]. Secondly, to ensure the mechanical integrity of the electrode, a large amount of insulating polymer binder needs to be introduced in the process, which not only increases the ineffective “dead volume” and “dead mass” but also hinders electronic conduction and ion migration, thereby significantly reducing the overall energy density and power density of the device [6]. Furthermore, such processes are limited to constructing two-dimensional planar structures, greatly restricting the design freedom of devices and making it difficult to meet the growing demand for complex configurations and space-efficient integration in emerging applications such as wearable electronics and miniaturized implantable medical devices [7]. Therefore, breaking away from the constraints of traditional electrode manufacturing paradigms and pursuing synergistic innovation in both “materials” and “structures” to construct new types of electrodes with precisely controlled three-dimensional architectures is a crucial pathway to addressing the current bottlenecks in electrochemical energy storage technology.

### 1.2. 3D Printing Technology: A Paradigm Shift for Electrochemical Energy Storage Design

3D printing, also known as additive manufacturing, is a disruptive fabrication technology that constructs three-dimensional objects layer by layer based on digital models [8]. Its “bottom-up” manufacturing philosophy highly aligns with the intrinsic need for complex three-dimensional structures in electrochemical energy storage devices, offering a novel technical pathway to overcome the limitations of conventional manufacturing methods. This technology offers exceptional freedom in structural design, enabling precise control over the spatial arrangement of materials and the programmable construction of complex 3D architectures, such as interdigitated, spiral, and bio-inspired hierarchical porous structures, spanning from the micro- to the macro-scale. These structures facilitate efficient transport pathways for electrons and ions, significantly optimizing the electrode’s internal transport kinetics [9,10]. Moreover, through multi-material printing or functionally graded material design, 3D printing enables the integrated fabrication of active materials, conductive agents, and solid electrolytes, thereby reducing interfacial impedance and allowing for synergistic customization of performance and structure [11]. It is particularly noteworthy that extrusion-based printing techniques (e.g., direct ink writing) rely on the inherent rheological properties of materials, enabling binder-free or binder-limited manufacturing, which effectively reduces inactive material content and enhances overall energy density [12]. Ultimately, this technology also facilitates the easy customization and miniaturization of devices, breaking the limitations of traditional two-dimensional form factors and precisely adapting to application scenarios with stringent requirements for unconventional shapes and spatial efficiency, such as wearable and implantable devices, thus opening new avenues for the design of next-generation energy storage systems [13,14].

Among the various 3D printing technologies available for fabricating energy storage devices, such as vat photopolymerization, powder bed fusion, and inkjet printing, extrusion-based direct ink writing has emerged as the most prominent technique for manufacturing carbon-based electrochemical energy storage devices. This is due to its excellent compatibility with high-solid-content, high-viscosity pastes (inks), relatively low cost, and broad material adaptability [15].

### 1.3. Carbon Materials: The Cornerstone of 3D-Printed Electrochemical Energy Storage

**Figure 1:**
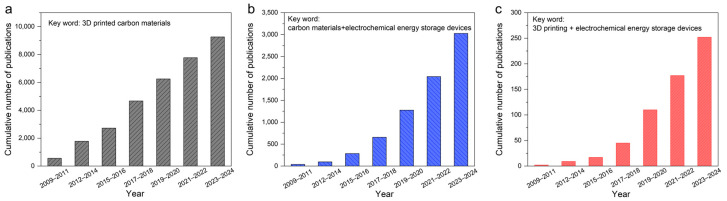
Cumulative number of publications related to 3D printing carbon-based electrochemical storage devices. Data were collected from the Web of Science for the years 2009–2024. Keywords: (**a**) “3D printed carbon materials,” (**b**) “carbon materials,” and “electrochemical energy storage devices,” and (**c**) “3D printing” and “electrochemical energy storage devices.”.

Carbon materials, known for their high electrical conductivity, excellent chemical/electrochemical stability, abundant availability, tunable pore structures, and surface chemistry, have always been indispensable in the field of electrochemical energy storage [16]. In the context of 3D printing, carbon materials play dual or even multiple key roles: they can serve as active materials (e.g., activated carbon, graphene, and carbon nanotubes) for building electric double-layer capacitive electrodes [17]; they can act as highly conductive three-dimensional current collectors or structural scaffolds (e.g., graphene aerogels and carbon nanotube sponges) for hosting other active materials (such as silicon, sulfur, and metal oxides), effectively mitigating volume changes during electrochemical processes while providing efficient electron conduction paths [18,19]; additionally, carbon materials (especially nano-carbons like graphene oxide) form colloidal suspensions with excellent rheological properties. By adjusting parameters such as concentration and pH, these suspensions can meet the stringent requirements of direct ink writing in terms of viscoelasticity, yield stress, and shear-thinning behavior, making them ideal matrix materials for formulating 3D printing inks [20,21]. Therefore, the combination of 3D printing and carbon materials can be regarded as a “perfect match” and is receiving increasing attention (Figure 1). Three-dimensional printing enables carbon materials to assume precise and complex macroscopic forms and intricate microscopic structures, while functionally diverse carbon materials provide 3D printing with high-performance ink systems and versatile application platforms. This interdisciplinary fusion is rapidly becoming a frontier in advancing the development of next-generation high-performance, customizable electrochemical energy storage devices.

Despite the significant potential demonstrated by 3D-printed carbon-based electrochemical energy storage devices, their transition from laboratory research to industrial-scale manufacturing faces several critical challenges, which also constitute the core scientific questions in this field. In terms of material design, there is an urgent need to develop high-performance carbon-based inks that combine excellent printability (rheological properties), structural stability (formability), and outstanding electrochemical performance. Research is also required to explore the synergistic effects of combining multiple nanocarbon materials (e.g., graphene oxide and carbon nanotubes) or compounding them with other active nanomaterials to enhance ink functionality and the performance of printed structures [22,23]. On the structural engineering front, it is essential to systematically establish the structure-property relationships between printing parameters (e.g., nozzle diameter, printing speed, layer height), ink composition, and the final printed structure’s attributes (such as resolution, porosity, and pore channel orientation). Furthermore, computer-aided design and artificial intelligence methods should be employed to optimize ideal three-dimensional structures for specific applications (e.g., high energy density, high power density, or mechanical flexibility) [24,25]. The core of this method lies in constructing an efficient “structure-performance” prediction model and using computational aids to guide experimental design and performance optimization. Specifically, the research first identified key structural parameters as design variables, such as electrode thickness, gap distance, and geometric area. A parameter space containing multiple potential combinations was constructed through computer-aided design, and representative experimental points were selected from it as training data for the machine learning model. Different algorithms were used to predict the capacitance performance. This significantly improved the efficiency and accuracy of electrode development, breaking through the limitations of traditional trial-and-error methods in terms of time and resources, and providing a powerful tool for the development of next-generation high-performance and customizable electrochemical energy storage devices. Post-processing techniques are equally critical. Processes such as thermal reduction, freeze-drying, and supercritical drying significantly affect the chemical properties (defects, functional groups), electrical conductivity, and mechanical strength of the printed carbon structures. Thus, developing milder and more efficient post-processing technologies is an essential current research focus [26,27]. Notably, adding fillers (such as silica and nanowires) to the ink can also enhance the mechanical stability of printed devices to a certain extent [28,29]. Finally, in the area of device integration and performance evaluation, achieving fully 3D-printed devices (integrating electrodes, separators, and encapsulation) remains challenging. There is a need to further elucidate the quantitative relationship between microstructural parameters and macroscopic electrochemical performance (e.g., volumetric energy density and areal capacity), and to systematically evaluate the devices’ long-term cycling stability and mechanical reliability [13,30].

**Figure 2:**
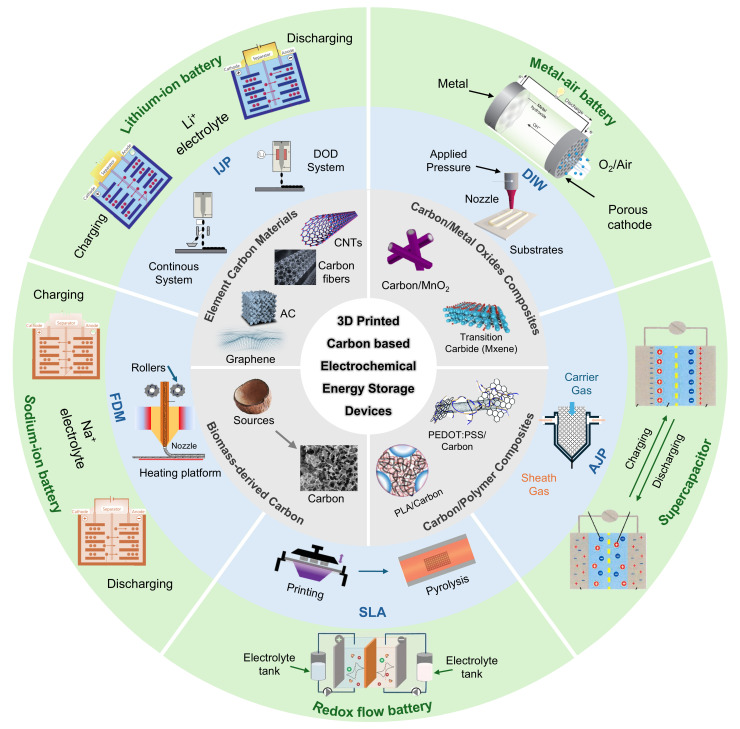
Schematic illustration of 3D-printed carbon-based electrochemical energy storage devices, from inside out, representing carbon materials, 3D printing technologies, and devices.

This review aims to systematically summarize recent research advances in 3D-printed carbon-based electrochemical energy storage devices (Figure 2). It will first introduce the various carbon materials and explore the material design principles of carbon-based inks, analyzing preparation strategies, rheological property regulation, and functional modification of different types of carbon inks. Then, it will review breakthroughs in diverse application scenarios, including high-areal-capacity devices, high-power devices, and miniaturized devices. Finally, we will outline current challenges and future development directions in this field, hoping to provide valuable insights and inspiration for researchers engaged in this interdisciplinary area.

## 2. Classification and Preparation Technology of 3D Printing Carbon-Based Materials

To achieve high-performance electrochemical energy storage, it is crucial to construct carbon-based electrodes with both excellent intrinsic properties and precise three-dimensional microstructures. This section aims to systematically elaborate on the full-chain preparation technology of carbon-based materials for 3D printing. Firstly, the carbon-based material system is the cornerstone of performance. This section will provide a comprehensive review of the intrinsic properties of elemental carbon materials and carbon-based composites, laying the groundwork for ink preparation and technology selection. Subsequently, the design of carbon-based inks suitable for 3D printing is the key bridge to achieving molding. It will focus on how to convert functional carbon materials into printable inks by regulating parameters such as rheological properties. Finally, 3D printing technology is the means of realization. This section will compare and analyze the principles and characteristics of direct writing printing, inkjet printing, aerosol jet printing, stereolithography apparatus, and fused deposition modeling, clarifying their compatibility with different carbon-based inks and the characteristics of the structures they construct. The three aspects progress step by step, jointly forming a complete technical roadmap from material selection, ink optimization, to technology implementation, providing a solid foundation for the customized design of three-dimensional carbon-based electrodes.

### 2.1. Carbon-Based Materials

#### 2.1.1. Elemental Carbon Material

##### Graphene

Since its discovery in 2004 [31], graphene has become a star two-dimensional material in fields such as flexible electronics [32], energy storage [33], and biomedical engineering, thanks to its single-layer sp^2^ carbon network that endows it with an ultra-high electron mobility (~2 × 10^5^ cm V^−1^ s^−1^), room-temperature quantum Hall effect, high mechanical strength (Young’s modulus ~1 TPa, fracture strength 130 GPa), high thermal conductivity (>3000 W m^−1^ K^−1^), and 97.7% optical transmittance [34,35,36,37]. However, its hydrophobicity leads to difficulties in dispersion and layer-by-layer stacking, which limits macroscopic molding [38]. High-concentration GO or rGO ink can be prepared through strategies such as oxidation-reduction, liquid-phase exfoliation, and electrochemical exfoliation, and then combined with 3D printing technologies such as inkjet writing, FDM, and EHD jetting to precisely construct micro-nano multi-level porous structures under low-temperature and mold-free conditions [39,40]. Coupling the two-dimensional structural advantages of graphene with the spatial forming ability of 3D printing can bring a new paradigm of simultaneous improvement in energy and power density for electrochemical energy storage devices.

##### Carbon Nanotubes (CNTs)

CNTs possess a one-dimensional hollow graphite lattice structure, featuring an ultra-high aspect ratio (>1000), a theoretical specific surface area of over 1000 m^2^ g^−1^, an axial Young’s modulus of approximately 1 TPa, an electrical conductivity as high as 10^5^ S m^−1^, and excellent chemical/mechanical stability, making them ideal electrode materials in the field of electrochemical energy storage [41]. Oriented CNT arrays can form continuous and low-curvature electron/ion transport channels, enhancing the charge mobility by over 40 times compared to random networks [42]. Additionally, their tunable pore size and surface chemistry enable the construction of hierarchical porous networks, which significantly reduces ion diffusion resistance and increases the utilization of active sites [43]. Among CNTs, multi-walled carbon nanotubes (MWCNTs) have received particular attention due to their excellent electrical, mechanical and thermal properties. The high electrical conductivity of MWCNTs has been utilized in various innovative ways to enhance the conductivity of nanocomposites, thereby achieving autonomous load sensing capabilities based on the piezoresistive effect. The manufacturing of miniaturized and stretchable devices through 3D printing provides low internal resistance, high power, and long-life solutions for flexible and wearable micro-energy storage systems.

##### Active Carbon (AC)

Activated carbon materials possess a high specific surface area, a rich multi-level pore structure (coexistence of micropores, mesopores, and macropores), good electrical conductivity, and excellent chemical stability. These properties make them ideal electrode materials for electrochemical energy storage devices such as electric double-layer capacitors (EDLCs) [44,45]. The high specific surface area provides a large number of charge adsorption sites, and the multi-level pore structure facilitates the rapid transport and diffusion of electrolyte ions, thereby enhancing the power density and rate performance of the device. In addition, the pore structure and specific surface area of activated carbon can be further regulated through chemical activation (such as CO_2_ activation), reaching up to 2200 m^2^ g^−1^, which significantly enhances its capacitance performance [46]. Its good mechanical flexibility and processability also make it suitable for flexible electronic devices and 3D printing technology, enabling the precise manufacturing of complex structures and further expanding its application prospects in next-generation high-performance and customized energy storage systems.

##### Carbon Fiber

Carbon fiber materials exhibit extremely high specific strength and specific modulus, as well as excellent electrical conductivity and good chemical stability, with a controllable multi-scale structure [47,48,49]. Carbon fiber has high electrical conductivity and can be used as an ideal electrode material or current collector, significantly reducing the internal resistance of devices and improving the charging and discharging efficiency. Its high specific surface area and controllable pore structure are conducive to the penetration of electrolytes and ion transport, enhancing power density and rate performance. In addition, carbon fiber composite materials can be integrated into complex structures through technologies such as 3D printing, including multi-layer electrodes and interwoven conductive networks, further optimizing the electrochemical interface and mechanical stability [50]. These characteristics make carbon fiber materials particularly suitable for advanced electrochemical energy storage devices such as high-performance supercapacitors [51], lithium-sulfur batteries [52], and structure-function integrated energy storage systems [53], providing an important material basis for achieving the next-generation energy storage systems that are lightweight, have high energy density, and long cycle life.

In conclusion, elemental carbon materials, with their diverse structures and excellent properties, have demonstrated great application potential in 3D-printed electrochemical energy storage devices. Graphene, with its unique two-dimensional structure and extremely high electrical conductivity, mechanical strength, and thermal conductivity, is suitable for constructing efficient conductive networks. Carbon nanotubes, with their one-dimensional hollow structure and ultra-high aspect ratio, significantly optimize the electron/ion transport path. Activated carbon, with its extremely high specific surface area and rich multi-level pore structure, has become an ideal choice for double-layer capacitors. Carbon fiber stands out due to its high specific strength, high modulus, and adjustable porous structure, making it suitable for high-performance electrodes and structure-function integrated devices. To more clearly demonstrate the core performance differences among the four types of materials, the following comparison is made from key indicators such as specific surface area, electrical conductivity, and mechanical strength (Table 1). Different elemental carbon materials have their own emphases in electrochemical energy storage. Through 3D printing technology, their intrinsic properties can be fully utilized to achieve multi-scale and multi-structure synergistic electrode design and manufacturing, thereby promoting the development of next-generation high-performance, customized energy storage systems.

#### 2.1.2. Carbon-Based Composite Materials

##### Carbon/Metal Oxides Composites

Carbon/metal oxide composites have demonstrated great application potential in the field of electrochemical energy storage due to their unique structural and performance advantages [54]. These materials combine high-conductivity carbon materials (such as activated carbon, carbon nanotubes, graphene, and other carbon-based materials) with metal oxides of high theoretical capacity, effectively integrating the mechanisms of double-layer capacitance and pseudocapacitance, and significantly enhancing the overall performance of the electrodes. Among the metal oxides, MnO_2_ has become the most widely used material because of its high theoretical specific capacitance (1370 F g^−1^) [55], abundant reserves [56], simple preparation process, and eco-friendly properties [57]. Herein, we will introduce several common types of these composite materials.

##### Activated Carbon/MnO_2_ Composites

Activated carbon/MnO_2_ composites are low-cost with simple preparation and have a large specific surface area. This composite can synergistically improve the capacitance of active carbon and the conductivity of MnO_2_, and enhance the wettability of the electrode surface, making it easier to adsorb electrolyte ions [58]. For example, Choi et al. reported a type of activated carbon/MnO_2_ composite electrode material, realizing a specific capacitance of 60.3 F g^−1^, and after 5000 cycles, the capacitance retention remained 99.6% [59].

##### Carbon Nanotube/MnO_2_ Composites

**Figure 3:**
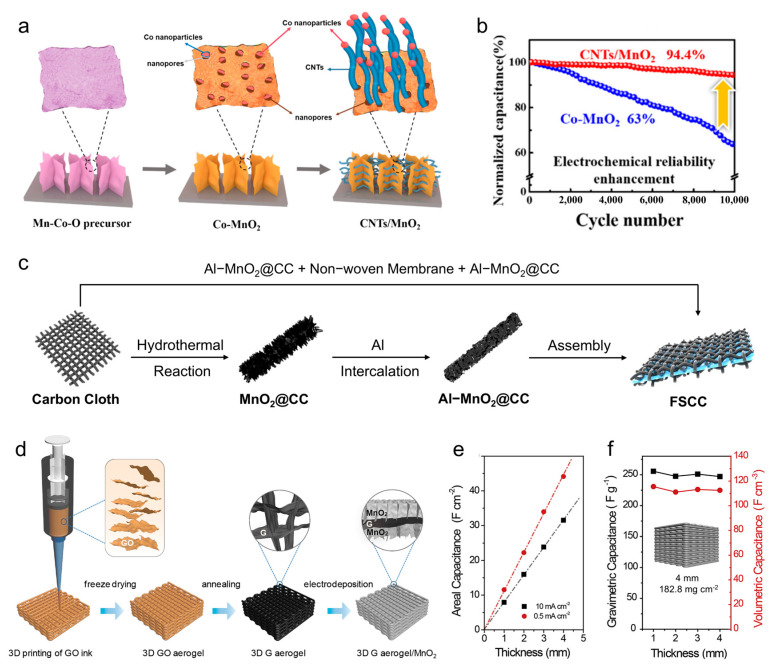
(**a**) Fabrication process of 3D-printed CNTs/MnO_2_ composite. (**b**) Cycling performance of a supercapacitor [62], copyright 2018, American Chemical Society. (**c**) Schematic illustration of 3D-printed CNTs/MnO_2_ composite, ref. [63] copyright 2020, Springer Nature. (**d**) Schematic of fabrication of a 3D-Printed Graphene Aerogel/MnO_2_ Electrode. (**e**) Areal capacitance of the electrodes measured at 0.5 and 10 mA cm^−2^ is plotted as a function of electrode thickness. (**f**) Gravimetric capacitance and volumetric capacitances are plotted as a function of electrode thickness, copyright [64], copyright 2019, Elsevier.

Carbon nanotube/MnO_2_ composites can combine the excellent electrical conductivity and mechanical properties of CNTs with the high specific capacitance of MnO_2_. MnO_2_ can improve the effective specific surface area and increase chemically active sites of the composites, leading to more excellent electrochemical properties [60]. For instance, Lei et al. prepared a CNTs/MnO_2_ composite via chemical vapor deposition and electrodeposition. This composite manifested a high specific capacitance of 615.6 F g^−1^, and the capacitance retention remained 95% after 5000 cycles [61]. To obtain a higher electrical chemical property, Jia et al. designed a CNTs/MnO_2_ composite with high MnO_2_ content (>90 wt.%) (Figure 3a) [62]. This composite realized a high specific capacitance of 1131 F g^−1^, and the capacitance retention still maintained 94.4% after 10,000 charge–discharge cycles (Figure 3b).

##### Graphene/MnO_2_ Composites

As a typical two-dimensional carbon material, graphene exhibits extraordinary mechanical properties, electrical conductivity, and a reasonably high specific surface area. Integrating with MnO_2_, the Graphene/MnO_2_ composites combine the above superiority of graphene with the pseudocapacitive properties of MnO_2_, constructing a three-dimensional conductive network with an enhanced electron migration rate and improved electrochemical performance [65]. Xiong et al. developed a Graphene/MnO_2_ material via thermal reduction, achieving a specific capacitance of 266.75 F g^−1^, with capacitance retention of 90% after 1000 cycles [66]. Additionally, reduced graphene oxide (rGO) is considered a promising material, which can be attributed to its low cost and high performance. Wang et al. employed a rGO/MnO_2_ composite via a hydrothermal approach, achieving a higher specific capacitance of 324.8 F g^−1^, and the capacitance stability was still maintained at 90.3% after 3000 cycles [67].

##### Other Carbon Materials/MnO_2_ Composites

Apart from the above-mentioned carbon materials, other carbon materials, including zero-dimensional materials (carbon quantum dots, nanospheres), one-dimensional materials (carbon nanofibers, nanowires), two-dimensional carbon cloth, and three-dimensional aerogel-like carbon materials, reveal unique superiorities in ion conduction, mechanical toughness, and flexible support. For instance, while carbon quantum dots combine with MnO_2_, the composites behave decent hydrophilicity, low toxicity, and can be easily functionalized, making them a suitable material for supercapacitors [68]. Carbon nanofibers have good electrical conductivity (~10^5^ S m^−1^) and stability. But its dense structure makes it behave with low porosity, resulting in unsatisfactory electrochemical properties [69]. With the help of combining them with MnO_2_, the electrochemical performances of Carbon nanofibers/MnO_2_ composites can be increased effectively. Compared to carbon nanofibers, the two-dimensional carbon cloth possesses a larger specific surface area, better electrochemical and mechanical properties, and unique flexibility, which render it an ideal material for a flexible electrochemical energy storage device. For example, Xu et al. synthesized a composite using Al-doped MnO_2_ and carbon cloth (Figure 3c), showing a specific capacitance of 1043 mF cm^−2^ and a capacitance stability of 91.1% after 5000 cycles [63]. The composite was then prepared to form a flexible, symmetric supercapacitor, exhibiting decent electrochemical performance and bending mechanical properties. Furthermore, the three-dimensional aerogel-like carbon materials (such as graphene aerogel and graphene hydrogel), which have abundant porosity and low density, are promised to be alternatives for supercapacitor electrode materials. Yao et al. developed a 3D Graphene aerogel/MnO_2_ composite (Figure 3d), exhibiting a specific capacitance of 44.13 F cm^−2^ with a MnO_2_ loading of 188.2 mg cm^−2^ (Figure 3e) [64]. In comparison to traditional electrode materials, this type of composite generally displays outstanding area, weight and volume normalized capacitance, proposing a strategy for designing high performance composite materials for electrochemical energy storage devices (Figure 3f).

##### Transition Metal Carbide

**Figure 4:**
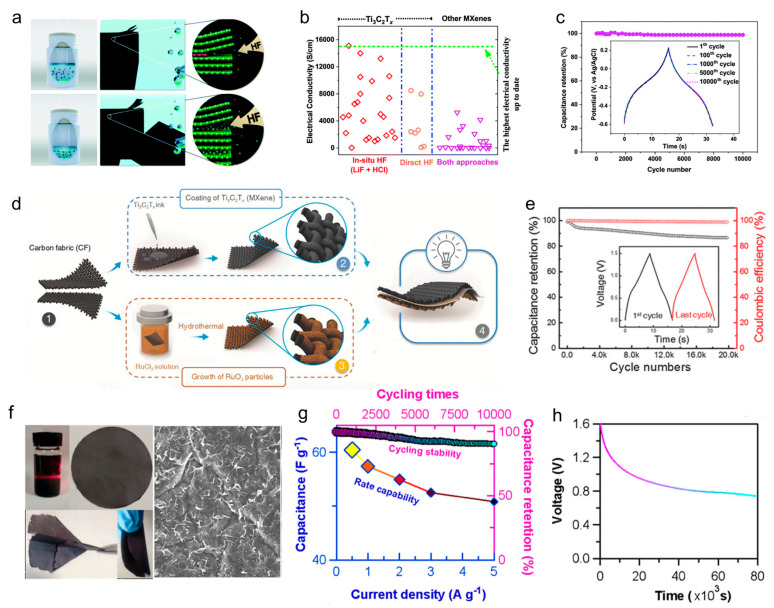
(**a**) Schematic of an optimized etching process for Mxene preparation. (**b**) Electrical conductivity of Ti_3_C_2_T_x_ MXene and other MXenes, and (**c**) Capacitance retention test of the prepared MXene film performed by galvanostatic cycling at 20 A g^−1^ [72], copyright 2019, Royal Society of Chemistry. (**d**) Schematic illustration of the fabrication process of the asymmetric Ti_3_C_2_T_x_ MXene formed supercapacitor. (**e**) Cycling stability and coulombic efficiency of the asymmetric device over 20,000 cycles at a current density of 20 A g^−1^ [73], copyright 2018, Wiley-VCH. (**f**) Digital photographs of Ti_3_C_2_T_x_ MXene colloid and flexible films, and SEM images of the Ti_3_C_2_T_x_ MXene film surface. (**g**) Current density-dependent specific capacitance change and cycling time-dependent capacitance retention change plots, and (**h**) self-discharge curve of the device, ref. [74] copyright 2021, American Chemical Society.

In recent years, a new type of carbon material, Mxene, has been developed. This is a two-dimensional material proposed by Gogosti in 2011 [70]. It is usually composed of transition metal carbides. Mxene has shown excellent application prospects in various fields, especially in the field of energy storage, due to its high conductivity, high electronegativity, high specific surface area, and good surface hydrophilicity [71]. Meanwhile, the abundant functional groups on the surface of MXenes enable them to have excellent dispersibility in polar solvents, giving MXenes a natural advantage during the process of being processed into conductive inks for 3D printing. And researchers have already achieved an electrical conductivity of up to 24,000 S cm^−1^ of Mxene by optimizing the etching process (Figure 4a) [72]. This promised MXene ink a strong candidate to replace traditional metal-based conductive inks and was expected to promote its application in electrochemical energy storage devices and conductive materials.

As a typical representative, Ti_3_C_2_T_x_ MXene possesses a high specific surface area and excellent ionic transport properties [75], and is currently one of the most widely studied materials used as electrodes in supercapacitors (Figure 4b,c). Jiang et al. designed a fully pseudo-capacitive asymmetric supercapacitor by combining MXene with a ruthenium oxide cathode (Figure 4d) [73]. The working window of this device was extended to 1.5V, which was approximately twice that of the symmetric MXene supercapacitor. Notably, carbon fibers were uniformly coated on the Ti_3_C_2_T_x_ MXene sheets. This supercapacitor exhibited extremely high stability, with its capacitance remaining at around 86% after 20,000 charge–discharge cycles (Figure 4e). Xu et al. combined the multi-electron redox reversible and structurally stable indoleketo π-framework with reduced graphene oxide to form an IDT@rGO molecular heterojunction as the positive electrode material (Figure 4f). Mxene films were used as the negative electrode material, and a flexible supercapacitor was constructed with IDT@rGO. The supercapacitor displayed a capacitance of 60 F g^−1^ (Figure 4g), and its output voltage could reach 1.6 V (Figure 4h). This device also had excellent cycle stability and mechanical flexibility [74].

##### Carbon/Polymer Composites

**Figure 5:**
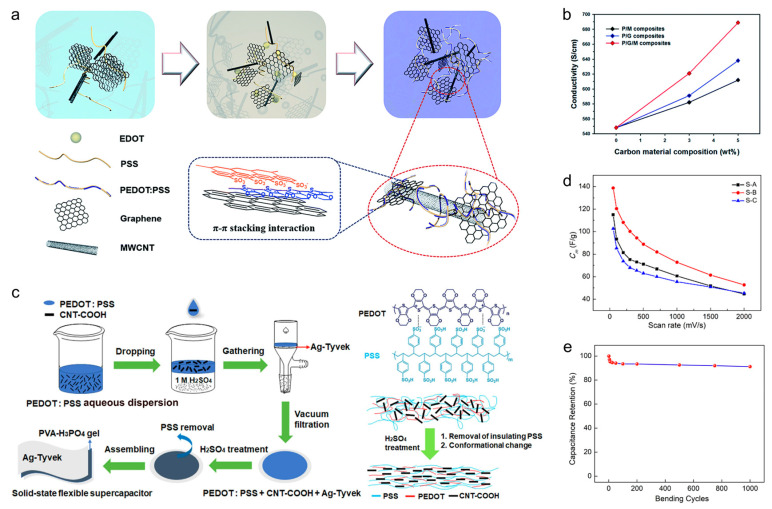
(**a**) Schematic of the synthesis of PEDOT:PSS/graphene/MWCNT (P/G/M) composites, and (**b**) electrical conductivity of the composites, ref. [78] copyright 2013, Royal Society of Chemistry. (**c**) Schematic of preparation of the flexible supercapacitor based on the Tyvek/Ag/PCNTs electrodes, and (**d**) volumetric capacitance. (**e**) Capacitance retention of the device under 1000 bending cycles, ref. [79] copyright 2021, Elsevier.

As is known, polymers generally have the advantages of being solution processable, good mechanical properties and can be easily composited. Among these materials, conductive polymers such as polypyrrole (PPy), polyaniline (PANI), and poly(3,4-ethylenedioxythiophene)–Polystyrene sulfonate (PEDOT:PSS) are suitable candidates for electrode materials due to their high specific capacitance, high electrical conductivity, and reversible redox performance. However, electrodes composed of these polymers generally lack specific capacitance stability, which limits their further application [76,77]. By the way of compounding with carbon materials, carbon/polymer composites can perform remarkable electrochemical properties with excellent cyclic stability. For instance, Yoo et al. synthesized a ternary composite using PEDOT:PSS as a matrix, which was hybridized with graphene sheets and multi-walled carbon nanotubes (Figure 5a) [78]. This composite displayed an electrical conductivity of 689 S cm^−1^, which was approximately 26% higher than that of pristine (Figure 5b). Under the duplicate total content of carbon materials, the electrical conductivity of the ternary composite material was 8% and 13% higher than that of the PEDOT:PSS/Graphene and PEDOT:PSS/carbon nanotubes composite materials, respectively. This research demonstrated that conductive polymers (PEDOT:PSS) and carbon materials existed tough electronic interactions, which could build up an excellent electrical bridge between conductive domains. As a modification of the PEDOT:PSS/carbon materials composite, Liu et al. used Ag-coated Tyvek paper as a substrate and fabricated an Ag-coated Tyvek/PEDOT:PSS/carbon nanotubes composite to prepare a supercapacitor (Figure 5c), realizing a specific mass capacitance of 138.7 F g^−1^ (Figure 5d). The substrates acted as the current collectors and endowed the supercapacitor with brilliant stability, rate capacity, and strong mechanical and flexible properties (Figure 5e) [79]. Abshirini et al. fabricated a polydimethylsiloxane (PDMS)/MWCNTs nanocomposite strain sensor with high tensile strength and conductivity using stereolithography printing technology [80]. Due to the excellent electrical conductivity, mechanical strength, and flexibility of MWCNTs, along with their ease of dispersion in PDMS, the fabricated devices exhibit excellent piezoresistant response, with an average gauge factor of 4.3. They also possess outstanding long-term stability, showing a decrease of only 2.6% after 300 cycles.

In addition, carbon materials-reinforced polymer composites are another widely studied type of carbon/polymer composites. Among them, carbon fiber (including recycled carbon fiber and continuous carbon fiber) reinforced polymer composites are broadly applied because of their tough mechanical strength and high stiffness-to-weight ratio [81]. Particularly, the recycled carbon fiber, which might be obtained from carbon fiber wastes, reveals superiority in reducing costs and protecting the environment [82]. And it has been demonstrated that 3D printing technology is an attractive way to manufacture the recycled carbon fiber reinforced polymer composites, which can be used to fabricate complicated electrochemical energy storage devices with high performance [83].

##### Biomass-Derived Carbon-Based Materials

Biomass is a natural, abundant, renewable, and carbon-rich resource, which can serve as an effective carbon source for fabricating kinds of carbon materials. Biomass-derived carbon-based materials possess high conductivity, large specific surface area, excellent chemical stability, and can be easily prepared. These advantages make the composite materials applicable in many fields [84,85,86]. Herein, we mainly classify the biomass materials into cellulose, hemicellulose, and lignin. Cellulose usually possesses excellent mechanical properties, and hemicellulose has branching structures (including main chain, side chain, and branch chain). Lignin is a multi-ring macromolecule containing negatively charged groups and has a strong affinity for high-valent metal ions.

The properties of carbon materials derived from biomass materials are closely related to their synthesis strategies. Therefore, it is imperative to develop methods for preparing biomass-derived carbon-based materials with a high specific surface area and a high porosity structure. The thermochemical process is a frequently used method to derive carbon materials from biomass. Specifically, it refers to the process of thermal decomposition of organic substances under anaerobic conditions and in a high-temperature environment. At present, the commonly used thermochemical processes mainly include activation (chemical activation [87], physical activation [88], and self-activation [89]), template method (hard template [90], soft template [91]), hydrothermal carbonization [92], and molten salt carbonization [93].

With the increasing demand for renewable materials, research on biomass-derived carbon materials serving as electrochemical energy devices, such as lithium-ion batteries (LIBs) and sodium-ion batteries (SIBs), has been widely carried out. Lv et al. developed a porous carbon material as an anode for LIBs from peanut shells via chemical activation [94]. This porous carbon exhibited excellent porous structure (large specific surface area) and electrical conductivity, contributing to improved electrochemical performances. While serving as the anode material, the peanut shells-derived carbon demonstrated a reversible capacity of 474 mAh g^−1^ at a current density of 1 A g^−1^. Apart from that, the biomass-derived carbon can act as a matrix to combine with metal oxides (MnO_2_). Jiang et al. fabricated carbon fibers using bamboo chopstick waste, which was further integrated with MnO_2_. The biomass-derived carbon fibers/MnO_2_ composites served as anode for LIBs, showing a reversible capacity of 710 mAh g ^−1^ at a current density of 0.2 A g^−1^ [95].

##### Prussian Blue/Carbon Based Materials

Prussian blue and its analogues are a class of hexacyanometalate materials with an open three-dimensional framework. These materials have attracted much attention due to their spacious ion channels and reversible multi-electron redox reactions (such as Fe^2+^/Fe^3+^), and they have high theoretical capacities while used as electrode materials for potassium-ion and sodium-ion batteries. However, their low intrinsic electronic conductivity and the easy formation of [Fe(CN)_6_] vacancies during crystallization have seriously restricted their capacity utilization and cycling stability.

To overcome these inherent deficiencies, the combination of Prussian blue with carbon materials has been proven to be an extremely effective strategy. The introduction of carbon materials (such as carbon nanotubes, graphene, graphite, etc.) builds a conductive network throughout the electrode, greatly facilitating the rapid transfer of electrons to the active sites of Prussian blue [96,97]. More importantly, carbon materials can serve as ideal nucleation substrates, guiding the uniform and controllable growth of Prussian blue crystals. This not only effectively reduces lattice defects and the content of crystalline water but also enhances the interface stability through π-π interactions or covalent bonding between the two, preventing the active substances from detaching during cycling. Additionally, the composite of carbon materials is conducive to optimizing the microstructure of the electrode, forming porous channels that are favorable for electrolyte infiltration and ion diffusion, thereby enhancing the rate performance.

Ma et al. synthesized a Prussian blue/RGO composite material through a one-step method, which was further ground with Ketjen black and carbon ink, forming a PRG printing ink [98]. Using the screen printing technique, they fabricated a flexible sensing array on a PET substrate. As a hydrogen peroxide sensor, this device exhibited a high linear response within the range of 0–10 mM, with a sensitivity of 31.65 μA mM^−1^ cm^−2^, a detection limit of 1.78 μM, and showed good selectivity, stability, and mechanical flexibility. Katic et al. utilized 3D printing technology to fabricate graphene electrodes and introduced oxygen-containing functional groups through electrochemical pretreatment, thereby enhancing their surface activity and electron transport performance [99]. Subsequently, Prussian blue nanoparticles were electro-deposited onto the electrode surface to form a 3DGrE/PB composite electrode. The prepared sensor exhibited a good linear relationship within the range of 0–700 μmol L^−1^, with a detection limit of sub-micromolar level, and had high accuracy and repeatability.

The combination of Prussian blue and carbon materials ingeniously integrates the former’s excellent ion storage/redox capabilities with the latter’s outstanding electrical conductivity and structural stability, synergistically enhancing the overall electrochemical performance of the composite materials. Future research could focus on developing new types of porous carbon carriers, exploring more environmentally friendly and efficient composite processes, and further expanding the application of such composite materials in flexible and wearable energy storage devices, promoting their practical application.

The above-mentioned carbon-based materials possess excellent electrochemical performance. However, their practical application potential largely depends on whether they can be efficiently and precisely processed into complex-structured functional devices. Therefore, transforming carbon-based materials into ink systems suitable for printing and processing becomes a crucial step in realizing their functionalization and device fabrication. Next, we will focus on the design strategies of carbon-based inks and review the scientific issues related to component regulation, rheological behavior, and stability.

### 2.2. Design Strategies for Carbon-Based Inks for 3D Printing

The investigation of carbon-based inks is considered a central driving force in advancing the development of next-generation high-performance and customized electronic devices and functional structures. Its significance lies in the integration of the exceptional electrical and mechanical properties of carbon materials, such as graphene, carbon nanotubes, and MXene, with the unparalleled capability of additive manufacturing to form complex structures and offer extensive design freedom [49,100,101]. This synergy opens new pathways for fabricating lightweight, flexible, and high-performance electrochemical energy storage devices. However, the core challenge in realizing this potential lies in the design and preparation of the inks, which is fundamentally determined by the intricate balance among printability, stability, and functionality [102]. This part centers on addressing this critical challenge.

#### 2.2.1. Ink Composition and Rheological Properties

The first consideration is the composition of the ink and its rheological properties [103]. The design strategy needs to be carefully balanced. The selection of solvent systems (environmentally friendly water-based and highly efficient organic-based dispersions), dispersants, and surfactants (which improve dispersion but may sacrifice conductivity), as well as the determination of solid content (which directly affects conductivity and viscosity), jointly determine the key rheological behavior of the ink. An ideal ink must exhibit significant shear thinning and thixotropy to ensure smooth flow under high shear in the print head and quickly regain viscosity after deposition, thereby maintaining structural accuracy and preventing leakage.

When designing the ink, the first consideration is the composition of the ink and its rheological properties [104]. This requires precise balancing of various aspects, including the selection of the solvent system, water-based or organic-based. On the one hand, water-based inks have the advantages of being environmentally friendly and allowing for easy post-processing. However, the water-based conditions can easily lead to the agglomeration of carbon materials (such as graphene, carbon nanotubes), resulting in poor ink stability. On the other hand, water-based inks usually require the addition of dispersants, such as polyvinylpyrrolidone (PVP), PEDOT:PSS, or surfactants like sodium dodecyl sulfate (SDS) or sodium dodecylbenzenesulfonate (SDBS), to improve the dispersion of carbon materials. In contrast, organic systems, including N-methyl-2-pyrrolidone (NMP), dimethylformamide (DMF), and tetrahydrofuran (THF), can more effectively disperse carbon materials, thereby avoiding agglomeration and functionalization of the carbon materials, and thus endowing the ink with better stability and more ideal conductivity. Nevertheless, these organic solvents are generally harmful to the environment and human health, and they can cause nozzle corrosion. At the same time, the above-mentioned organic solvents commonly have intense volatility, which may cause the concentration of the ink to change during the printing process. This would affect the uniformity of the printing to some extent and even cause clogging of the nozzle. Therefore, the selection of a solvent system must be determined based on a comprehensive consideration of multiple factors, including the type of carbon material, the printing technique, the nozzle material, and the requirements of the printed device.

The rheological properties include viscosity, shear thinning, thixotropy and yield stress, which are the key determinants for achieving efficient and high-precision printing. Firstly, the viscosity of the inks determines which printing technology can be suitable. For example, the viscosity of ink suitable for inkjet printing is usually 5–20 cP, with a surface tension of 25–35 mN m^−1^. The composition and solid content of the ink have a significant impact on viscosity. Secondly, shear thinning behavior is a crucial non-Newtonian fluid property. In carbon-based inks, the suspension composed of sheet-like particles (such as graphene) exhibits a more significant shear thinning effect in the low shear rate region (γ < 1 s^−1^) compared to spherical particles [105]. This behavior is highly beneficial for the printing process, as it can reduce viscosity in the high shear conditions within the nozzle to facilitate flow, and restore viscosity after deposition, which helps maintain the structural shape. Hoath et al. confirm that shear thinning can effectively inhibit the formation of satellite droplets, thereby improving the printing resolution [106]. Closely related to shear thinning is thixotropy, which refers to the ability of viscosity to change over time. For example, the non-covalent network structure formed by graphene oxide (GO) is a perfect example of ideal thixotropy [107]. It enables the ink to have a high storage modulus and good structural recovery ability, thereby achieving excellent printability and the construction of self-supporting structures. Furthermore, many high-concentration carbon-based inks (such as those with high loading of carbon nanotubes or GO suspensions) exhibit significant yield stress. This means that the ink behaves like a solid when at rest and requires a critical stress to start flowing. This property ensures that the printed fibers or layered structures can maintain their shape without collapsing, which is the basis for achieving 3D stacking manufacturing. Hence, the rheological properties of carbon-based inks constitute a complex interrelated system that requires coordinated design and precise control to maintain the inherent functional attributes of carbon materials (such as conductivity) while meeting the requirements of specific printing processes.

An ideal ink should exhibit significant shear thinning properties and thixotropy to ensure the smooth fluidity of the ink at the high shear rates of the printing nozzle. Meanwhile, the viscosity of the printing ink needs to be quickly restored to maintain the structural accuracy of the printed patterns and prevent excessive spreading. Therefore, the rheological properties of the ink need to be precisely controlled. To determine the key rheological behavior of the ink, researchers principally considered the types of dispersants and surfactants, as well as the solid content of the functional components of the ink. Dispersants and surfactants are mainly used to improve the dispersion of carbon materials and prevent their agglomeration in the ink [102]. They can directly affect the conductivity and viscosity of the ink, and are chiefly divided into ionic surfactants, non-ionic surfactants, polymer dispersants, and biopolymers. Ionic surfactants, including SDS, SDBS, cetyltrimethylammonium bromide (CTAB), etc., prevent the agglomeration of carbon materials through electrostatic repulsion. Non-ionic surfactants, such as the Triton X series, Tween series, and Pluronic, achieve stable dispersion of carbon materials through spatial steric hindrance. Polymer-based dispersants contain PVP, PEDOT:PSS, gum arabic (GA), etc., which can also serve as binders. Biopolymers such as silk fibroin (Silk Fibroin), serum albumin (BSA), chitosan, etc., have good biocompatibility. However, it is worth noting that the side effects of dispersants and surfactants may lead to reduced conductivity of the ink, introducing impurities, and thus have negative impacts on printing performance. Therefore, a balance still needs to be achieved between dispersion and conductivity.

#### 2.2.2. Ink Stability and Post-Processing Requirements

The stability of ink and the requirements for post-treatment are crucial for ensuring its transition from laboratory research to practical applications [108]. The ink stability is closely associated with the dispersibility of carbon materials. Thus, preventing the agglomeration and sedimentation of carbon nanoparticles is the priority in maintaining the stability of the ink.

To avoid the agglomeration of carbon materials, it is necessary to control the intermolecular forces between carbon material particles, which is generally described by the Derjaguin–Landau–Verwey–Overbeek (DLVO) theory [109]. Stable suspensions of carbon materials in inks can be achieved through various methods, such as mechanical treatments (including sonication, ball milling, stirring, etc.), dispersion in solvents, functionalization of carbon materials, and the addition of external surfactants.

The stable dispersion of carbon materials in solvents relies on identifying solvents that can render the carbon materials thermodynamically soluble. That is, solvents with a negative mixing free energy when combined with carbon materials need to be determined. In the previous section, we have already listed some common solvents. And the specific choice should be based on the characteristics of the materials when formulating the carbon-based inks. Regarding functionalization modification, we take the covalent functionalization of CNTs as an example, which is mainly realized by modifying the sidewalls or terminal surfaces of CNTs through covalent attachment of functional groups such as –COOH, –OH, and –NH_2_ [110,111,112]. The introduction of functional groups enhances the solubility of CNTs in the chosen solvents. However, this covalent modification can affect the π-electron configuration of CNTs. Specifically, the transformation from π (sp^2^) to σ (sp^3^) will occur, which in turn influences the electrical properties of CNTs [113].

Similarly, in the foregoing content, we also enumerated many typical surfactants. The dispersibility of ink depends on multiple parameters of the surfactant, including its concentration, chain length, head group size, Zeta potential, and molecular structure. With regard to a given initial concentration of carbon materials, the performance of the surfactant generally improves with an increase in surfactant concentration until an optimal level is achieved. Beyond this point, the performance deteriorates as the surfactant concentration continues to rise. The reason why the dispersion degree improves initially is that the surfactant covers more surface area of the carbon materials as the surfactant concentration increases. However, there exist limited adsorption sites on the surface of carbon materials, signifying that the adsorption will reach saturation at a certain concentration, which may cause reverse micellization [114]. This reverse micellization can promote the aggregation of carbon materials.

Additionally, the post-treatment process of the printed ink is also a crucial factor in ultimately achieving the expected electrochemical performance and mechanical function of electrochemical energy storage devices. Common ink sintering methods comprise thermal sintering, light sintering, microwave sintering, and plasma sintering. For some inks such as carbon black and CNTs composites, high-temperature carbonization or activation treatment is required to improve conductivity and mechanical strength. For instance, high-temperature (>800 °C) treatment can remove non-carbon elements, thereby improving the purity and conductivity of CNTs. And using activators, including CO_2_ and water vapor, to treat CNTs can increase the specific surface area and pore structure, which renders CNTs suitable for manufacturing supercapacitors [102,103].

At the same time, it is necessary to consider suppressing the coffee ring effect of the ink during the printing process, which involves specific printing processes such as the roughness and hydrophilicity/hydrophobicity of the substrate surface. Zhang et al. systematically studied the morphologies of colloidal droplets formed under different surface roughness conditions, illustrating that the coffee ring effect became more obvious while the substrate roughness increased [115]. This was because the rough surface can inhibit the backflow of capillary flow and hinder substances from cycling back to the center of the droplet. More details about printing will be elaborated in the subsequent sections.

In summary, the design and fabrication of carbon-based inks are complex projects that involve multiple disciplines, including materials science, colloid chemistry, and fluid mechanics. Only by systematically optimizing the composition to regulate rheological properties and complementing it with effective stability strategies and post-treatment processes can prepared ideal inks that simultaneously meet the requirements of high-precision printing and high-performance applications. Consequently, the promised prospect of 3D-printed carbon materials will come true in the field of advanced electrochemical energy storage devices.

### 2.3. 3D Printing Technologies

Compared with the traditional slurry coating method, 3D-printed electrodes exhibit significant differences in specific area capacitance and specific volume capacitance. Electrodes prepared by the traditional slurry coating method usually present a dense two-dimensional layered structure. Although they can achieve a relatively high specific area capacitance, their ion diffusion paths are long and the electrode tortuosity is high, resulting in relatively limited specific volume capacitance. Especially under high loading conditions, the problem of ion transport kinetics hysteresis becomes more prominent, severely restricting their performance in the thickness direction. In contrast, 3D printing technology, through precise design of the macroscopic structure of the electrode, can construct electrodes with 3D interconnected channels and low tortuosity paths. Although this ordered porous structure may result in a slightly lower specific area capacitance due to the lower local loading compared to traditional electrodes, it provides efficient channels for ion transport, significantly improving the wettability of the electrolyte and the migration rate of ions. Under the same overall active material loading, 3D-printed electrodes, with their porous framework, can achieve higher specific volume capacity and significantly enhanced rate performance.

The successful design of carbon-based ink has laid the foundation for its application in 3D printing. However, the realization of 3D-printed carbon-based electrochemical devices is highly dependent on the coordinated matching of ink performance and printing processes. Different printing technologies have significantly different requirements for the physical and chemical properties of inks (such as viscosity, shear thinning behavior, curing mechanism, etc.). Therefore, it is necessary to select appropriate forming strategies based on the ink properties and target device structures. Based on the clear design principles of the ink, we further conduct a systematic comparison and analysis of the current mainstream 3D printing technologies to reveal their applicability and limitations in the manufacturing of carbon-based electrochemical devices. In this section, we will introduce common 3D printing technologies in terms of their technical characteristics, applicable materials, advantages and limitations. These technologies include direct ink writing (DIW), inkjet printing (IJP), aerosol jet printing (AJP), stereolithography (SLA), and fused deposition (FDM).

#### 2.3.1. Direct Ink Writing (DIW)

Direct ink writing (DIW) is an extrusion-based additive manufacturing technology with a relatively straightforward principle [116]. The high-viscosity ink contained in a syringe is driven by pneumatic pressure or a screw mechanism and extruded through a nozzle, then deposited layer by layer onto a substrate following a pre-designed path to construct complex three-dimensional structures. Carbon-based inks suitable for DIW must possess high viscosity (ranging from 2000 to 1,000,000 cP) and exhibit significant shear-thinning behavior to facilitate smooth extrusion and rapid shaping. Additionally, the ink should demonstrate rapid solidification capability and good shape retention after deposition.

During the printing process, the nozzle is positioned very close to the substrate (approximately 0.1 mm), which makes it contact the substrate when the extruded ink accumulates at the nozzle into a sufficiently large droplet. Due to the surface tension, the ink wets the substrate and forms a stable meniscus between the nozzle and the substrate, enabling continuous and stable printing. The nozzle size determines the printing resolution and can currently reach several tens of micrometers. However, DIW requires exact control of the nozzle height. Deviation beyond the allowable range may cause the rupture of the meniscus, leading to printing failure. Therefore, the flatness of the printing substrate is critical.

**Figure 6:**
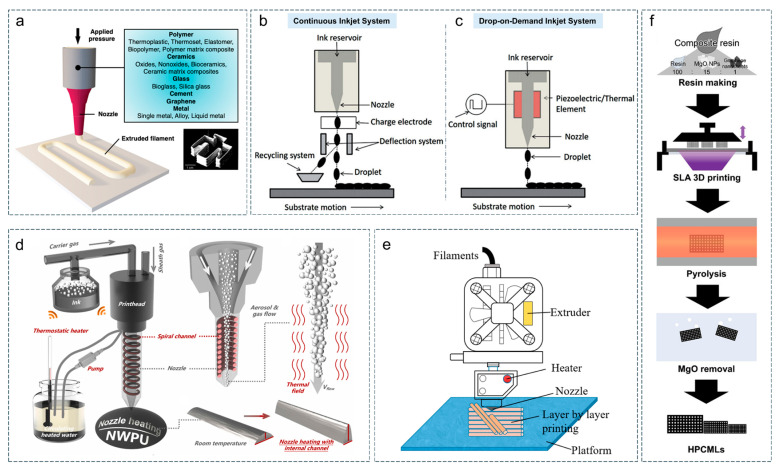
Schematic illustration of 3D printing technologies, (**a**) direct ink writing, ref. [117] copyright 2022, Wiley-VCH, (**b**) continuous inkjet system, (**c**) drop-on-demand inkjet system [118] copyright 2022, Wiley-VCH, (**d**) aerosol jet printing, ref. [119] copyright 2025, Elsevier, (**e**) fused deposition modeling [120] and (**f**) stereolithography apparatus, ref. [121] copyright 2023, Wiley-VCH.

This three-dimensional printing technology offers several advantages, including low cost, simple processing, and broad material compatibility. Virtually, any material that can be formulated into an ink can be printed using DIW (Figure 6a) [117]. Nevertheless, the technology also has some limitations. For instance, it struggles to achieve sub-micrometer resolution and has strict requirements for ink rheology. Otherwise, it is prone to cause nozzle blockage and poor printing continuity. The as-printed structures often exhibit low mechanical strength and usually require post-treatment to enhance stability.

In general, DIW is a versatile and precise three-dimensional manufacturing strategy that shows great promise in optimizing the architecture and enhancing the performance of electrochemical energy storage devices. For example, Zhang et al. prepared a Ti_3_C_2_T_x_ Mxene ink and printed conductive circuits, micro-supercapacitors, and ohmic resistors on paper substrates via DIW [122]. Due to the high electrical conductivity and pseudocapacitive properties of Mxene, the DIW-printed devices achieved higher volumetric capacitance and energy density.

#### 2.3.2. Inkjet Printing (IJP)

Inkjet printing is a non-contact, digitalized printing technology that precisely deposits functional ink in the form of droplets onto a substrate through micron-scale nozzles to form designed patterns or structures. This technology primarily includes continuous inkjet (Figure 6b) and drop-on-demand type (DOD) (Figure 6c), with the latter being more widely adopted due to its operational flexibility and precise droplet control [118]. DOD can be further divided into thermal and piezoelectric inkjet printing types. The piezoelectric method is particularly suitable for a broader range of material systems due to its independence from ink volatility and compatibility with high-temperature applications. The principle of piezoelectric inkjet printing involves applying an external voltage to induce a relative displacement of positive and negative charges within the piezoelectric crystal, resulting in its deformation. This deformation compresses the ink in the pressure chamber, ejecting it through the nozzle orifice to form droplets. By optimizing printer parameters such as driving voltage, pulse duration, and backpressure, the jetting velocity and droplet size can be precisely controlled, achieving a resolution finer than 100 μm.

Similarly, in terms of ink materials, IJP is widely compatible with various functional nanomaterial inks, including graphene, carbon nanotubes, and conductive polymers (e.g., PEDOT:PSS) [123,124]. These inks must exhibit low viscosity (5–20 cP), appropriate surface tension, and excellent stability to prevent particle aggregation and nozzle clogging. Furthermore, they are often formulated as colloidal dispersions in aqueous or organic solvents, frequently supplemented with surfactants or chemical functionalization to enhance dispersion stability and printability.

IJP eliminates the need for prefabricated templates or photolithographic masks, substantially reducing manufacturing costs and time, making it particularly suitable for rapid prototyping and small-batch customized production. It supports high-precision digital pattern control and enables multi-material and multi-layer printing. IJP is compatible with flexible, curved, and biocompatible substrates (such as paper, polyimide, and PET), making it applicable to emerging fields like wearable electronics and biosensors [123]. Additionally, it is environmentally friendly, with minimal material waste and low energy consumption, aligning with the requirements of sustainable manufacturing.

However, IJP also has certain limitations. Firstly, the coffee ring effect, which we have mentioned in the previous section, requires mitigation through solvent formulation optimization, substrate treatment, or temperature control [125]. Secondly, the printing resolution is constrained by the droplet size (typically 10–20 μm) and the repeatability of the printing position (±10 μm). Although resolutions can be improved to the submicron level using electrohydrodynamic (EHD) printing, this significantly increases system complexity. Thirdly, the technology demands extremely high ink dispersion stability, particularly for nanomaterials prone to agglomeration, such as CNTs. Ensuring ink uniformity often requires additional processing steps, such as ultrasonication, centrifugal purification, or chemical modification.

#### 2.3.3. Aerosol Jet Printing (AJP)

Aerosol jet printing was proposed in the early 2000s and later commercialized by the American company Optomec (St. Paul, MN, USA). When conducting aerosol jet printing, the ink is atomized and dispersed into liquid droplets with diameters ranging from 1 to 5 μm, which are then mixed with the transporting gas to form an aerosol [126,127,128]. The methods of atomization include ultrasonic atomization and pneumatic atomization. While operating, the atomized ink droplets are transported to the nozzle through the gas flow. To ensure that the aerosol droplets sprayed stably, the nozzle part is designed as a sandwich structure, which generates a of jetting gas flow (sheath gas) around the aerosol flow emitted from the nozzle (Figure 6d) [118]. This design implements gas dynamic focusing (aerodynamic focusing) on the atomized ink sprayed out, which keeps the aerosol flow within a range smaller than one-tenth of the nozzle diameter.

To the specific, AJP belongs to a continuous inkjet printing technology. The substance it sprays out is essentially a continuous gas flow containing a large number of microscopic ink droplets, rather than the traditional IJP, which only sprays a single independent ink droplet each time. AJP interrupts the spraying gas flow by using a barrier outside the nozzle, similar to the shutter of a camera. Therefore, the AJP does not print a dot matrix pattern composed of a large number of ink dots, but rather forms the required pattern through a series of continuous or intermittent lines.

Compared with traditional IJP, AJP has seen improvements in many aspects. Firstly, the viscosity range of the ink has been significantly expanded. AJP allows the ink viscosity to be within the range of 1 to 1000 cP. Low-viscosity ink (1–10 cP) can be atomized by ultrasound, while high-viscosity ink (10–1000 cP) can be atomized by pneumatic atomization. This expansion of the viscosity range greatly reduces the requirements for the printing ink and expands the material types used for ink. Secondly, the printing resolution is greatly improved. This is because the gas dynamics focusing effect renders the printing resolution no longer dependent on the nozzle diameter and ink droplet volume. Meanwhile, the droplet particles in the aerosol flow have a smaller diameter and a larger specific surface area, making their drying speed much higher than that of traditional IJP, which significantly reduces the fluidity of the ink droplets after reaching the substrate surface, and is also conducive to achieving high-resolution printing effects. Traditional IJP generally has difficulty completing a print line width of less than 20 μm, while AJP can achieve a print line width of less than 10 μm. Due to the gas dynamics focusing effect on the atomized ink, the working distance from the AJP nozzle to the substrate surface can be increased to 5 mm, which is much greater than 0.5 mm of IJP, and thus render it to print on surfaces with height differences within a certain range while maintaining the line width unchanged. In addition, on account of the significant increase in nozzle diameter, the carbon-based ink systems designed for AJP can contain larger solid particles.

Although AJP has significantly improved the precision of printing, its working principle has strict requirements for the stability of the air flow, which signifies that the air flow parameters need to be finely adjusted to ensure good printing results. At the same time, the droplet clusters sprayed by AJP will splash to a certain degree, which gives rise to the number of satellite points scattered outside the main body of the printed pattern being more than that of the IJP printed patterns.

#### 2.3.4. Stereolithography Apparatus (SLA)

Stereolithography apparatus is a process that selectively cures photosensitive resin with ultraviolet light, layer by layer, to form a three-dimensional structure. The solidification process involves scanning and curing the photosensitive resin from points to lines and then from lines to surfaces (Figure 6f) [121]. SLA has a relatively high level of forming accuracy, which can achieve a resolution of no less than 50 μm in all three spatial directions.

SLA demonstrates remarkable advantages in the preparation of carbon-based materials. For example, Kudo et al. reported a type of hierarchically porous carbon microlattices based on a composite photosensitive resin via SLA [121]. This composite resin was based on epoxy-phenolic resin, with magnesium oxide nanoparticles as pore-forming agents, and multi-layer graphene nanosheets were introduced to suppress ultraviolet scattering, thereby improving the printing accuracy and structural integrity. The SLA formed structures were carbonized by high-temperature pyrolysis, realizing a hierarchically porous structure comprising the lattice architecture (~100 μm), macropores (~5 μm), mesopores (~50 nm), and micropores (~1 nm). This SLA formed carbon micro-lattice possessed excellent electrochemical properties and can be used as a thick electrode for supercapacitors, which achieved a specific capacitance of 105 F g^−1^ in aqueous electrolytes and 13.8 F g^−1^ in organic electrolytes, with an areal capacitance exceeding 10 F cm^−2^ and 1 F cm^−2^, respectively. This demonstrates its potential for application in fabricating electrochemical energy storage devices.

Nevertheless, the printable materials are limited to photopolymer resins and their composite materials, and the dispersion and viscosity of additives need to be strictly controlled to avoid nozzle blockages or structural defects during the printing process. Moreover, during the pyrolysis process, there is still inevitable shrinkage and possible interlayer detachment, as well as incomplete bonding between carbon materials and the matrix, which may affect the mechanical properties and functional consistency of the final carbon devices.

#### 2.3.5. Fused Deposition Modeling (FDM)

Fused deposition modeling, proposed in 1988 by Scott Crump, is a rapid prototyping technology that achieves three-dimensional solidification through the high-temperature extrusion of thermoplastic materials and layer-by-layer stacking (Figure 6e) [120]. This technology not only has low manufacturing costs and simple operation, but also has favourable material adaptability and design flexibility [129]. It can produce structurally complex (printing resolution range from 50 to 200 μm) and highly integrated functional devices, and is particularly suitable for the development and prototype production of electrochemical energy storage devices, sensors, and customized experimental equipment.

In terms of the materials that can be processed, FDM can utilize various composite filaments containing conductive fillers. Among them, carbon black/poly(lactic acid) (CB/PLA) is currently one of the most common and economical conductive materials, and is widely used to fabricate electrochemical devices [130,131]. However, these materials typically exhibit limited conductivity and high charge transfer resistance when printed in their original state, severely restricting their electrochemical response performance. Nevertheless, numerous refinement methods (including electrochemical activation, mechanical polishing, and solvent treatment) have been employed to enhance their electrochemical properties.

The FDM 3D printing exhibits significant advantages in manufacturing carbon-based electrochemical devices, which lie in its rapid prototyping capability, customizable geometries, and potential for integrated molding. However, this technology is also confronted with some challenges, including weak interlayer bonding, high surface roughness, uneven distribution of conductive pathways, and other structural defects that can easily lead to unstable electrochemical performance.

Therefore, recent research has begun to focus on optimizing printing rather than post-processing. Rocha et al. reported that systematically regulating parameters such as printing direction, layer thickness, and printing speed can directly promote the ordered distribution of carbon black particles and the formation of conductive networks during the molding process [131]. Consequently, the electrochemically active area and electron transfer efficiency of the electrodes can be significantly enhanced, allowing for the fabrication of high-performance sensors without requiring post-processing. Therefore, the FDM technology provides strong support for the low-cost, short-cycle, and personalized manufacturing of carbon-based electrochemical devices. Combined with scientific parameters design and material engineering, it is expected to have broader application in the fields of sensing analysis and energy storage.

Additionally, it is worth noting that the technical parameters of 3D printing (such as nozzle diameter, printing speed, and layer thickness) have a significant impact on the performance of printed devices (such as structural accuracy, porosity, and surface roughness) [132]. The nozzle diameter is a key parameter that determines the minimum feature size of the printed structure. For instance, in DIW, the nozzle diameter can be tens of micrometers, but the actual printing resolution is limited by the rheology of the ink and the size of the particles. The ratio of the nozzle diameter to the maximum particle size of the filler should be greater than 100–150 to avoid clogging [133]. A smaller nozzle diameter helps form finer fiber structures, increasing the specific surface area and porosity of the electrode, which is beneficial for the diffusion of electrolyte ions. A micron-sized nozzle can be utilized to construct a 3D network structure with continuous channels. Undoubtedly, high-resolution printing can increase the active material loading and ion accessibility of the electrode, thereby enhancing the energy density and rate performance. The printing speed needs to be determined based on the material properties. For example, in FDM and DIW, the printing speed affects the spreading and interlayer bonding of the molten or extruded material. A speed that is too fast can lead to poor interlayer bonding and the formation of cracks; a speed that is too slow may cause excessive spreading and reduce structural accuracy. The layer thickness determines the longitudinal resolution of the printed structure. A smaller layer thickness (such as tens of micrometers in SLA) can enhance the interlayer bonding strength and reduce internal defects. In DIW, the layer thickness is closely related to the nozzle diameter and ink viscosity. A smaller layer thickness helps form a denser layered structure but may reduce the overall porosity; appropriately increasing the layer thickness can introduce more vertical channels and promote ion transport. Optimizing the layer thickness can improve the ion conductivity and rate performance of the electrode while maintaining structural strength. For example, when printing thick electrodes, controlling the layer thickness to construct a hierarchical pore structure can achieve a high area capacity [134]. Other parameters such as curing and sintering conditions, as well as post-processing techniques, also need to be considered.

This chapter focuses on the application of 3D-printed carbon-based materials in electrochemical energy storage, reviewing the types of carbon-based materials, ink formulations, and printing technologies. In terms of the materials system, we systematically summarize the characteristics of elemental carbon and various carbon composite materials, as well as their applications in electrodes. Subsequently, we expound on the design challenges and solution strategies of carbon-based inks in terms of rheological properties, stability, and post-processing adaptability. Finally, we compare and analyze the forming mechanisms, material compatibility, and structural shaping capabilities of mainstream 3D printing technologies, revealing the advantages and limitations when different technologies are combined with carbon-based inks. The entire chapter progresses step-by-step, forming a systematic methodology that encompasses conductive carbon-based materials, inks, and device fabrication processes, providing theoretical guidance and technical support for the controlled preparation of complex three-dimensional carbon-based electrochemical energy storage devices.

## 3. Applications in Typical Electrochemical Energy Storage Devices

Three-dimensional printing, through the precise design and controlled construction of material structures in three-dimensional space, provides unprecedented freedom for preparing high-performance batteries, supercapacitors, and other electrochemical devices. Regarding carbon-based materials, 3D printing not only enables the controlled preparation of complex hierarchically structured pores, optimizing the ion and electron transport paths, but also effectively increases the specific surface area and active material loading of the electrodes, thereby significantly improving the energy density and power density of the devices. Based on the above description of carbon-based materials, carbon-based inks, and common printing technologies, this section mainly introduces the application of 3D-printed carbon-based materials in lithium-ion batteries (LIBs), sodium-ion batteries (SIBs), metal–air batteries (MABs), redox flow batteries (RFBs), and supercapacitors (SCs).

### 3.1. Lithium-Ion Batteries (LIBs)

LIBs are currently the battery system with the best overall performance, possessing high specific energy, long cycle life, small size, light weight, and non-pollution. They have rapidly developed into a new generation of energy storage power sources and are widely used in information technology, electric vehicles, aerospace, and other fields [135,136]. To further enhance the electrochemical properties of LIBs, research is focused on developing new lithium storage materials and electrode materials. Three-dimensional printing technologies, particularly DIW and FDM, are widely used for printing carbon-based materials, including graphene, carbon nanotubes, and multi-walled carbon nanotubes, providing a new approach for the customized design and manufacturing of LIB electrodes. The printed carbon-based electrodes have demonstrated excellent results in various electrochemical performances, including high specific capacity, good rate performance, and long cycle life, providing an essential direction for the development of future high-performance lithium-ion batteries [137,138,139].

**Figure 7:**
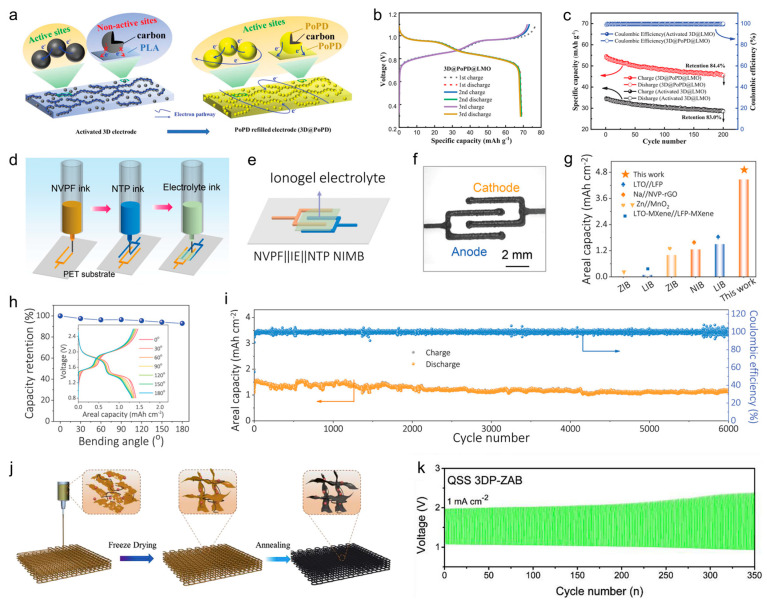
(**a**) Schematic of the charge transfer difference between the activated 3D-printed and PoPD-refilled carbon electrode. (**b**) GCD curves of the printed 3D@PoPD@LMO electrodes. (**c**) Comparison of cycling performance between activated 3D@LMO and 3D@PoPD@LMO electrodes, ref. [140] copyright 2021, Wiley-VCH. (**d**) Schematic of the aqueous NVPF, NTP, and ionogel electrolyte ink for 3D printing. (**e**) Photograph and (**f**) schematic of the printed NVPF||IE||NTP NIMB/EC. (**g**) Comparison of the areal capacity with the reported microbatteries. (**h**) Capacity retention as a function of bending angles. (**i**) Long-term cycling stability, ref. [141] copyright 2022, Wiley-VCH. (**j**) Schematic of the 3D-printed air electrode and (**k**) cycling performance of the printed ZAB, ref. [142] copyright 2020, Elsevier.

By means of FDM, Gao et al. fabricated a carbon framework using commercially available carbon black/PLA composite filament (carbon black < 21.43%). The carbon framework was treated with DMF to remove the surface PLA, and then modified poly(ortho-phenylenediamine) (PoPD) via electrodeposition (Figure 7a) [140]. Employing the PoPD-deposited frameworks with LiMn_2_O_4_ composites as the cathode, the LIB provided a specific capacity of 69.1 mAh g^−1^ at a specific current density of 0.036 mA cm^−2^ (Figure 7b), and the capacity retention remained at 84.4% after 200 cycles (Figure 7c). This study established a pathway toward fabricating electroactive 3D-printed electrodes using economical low-dimensional carbon-based materials, specifically for application in aqueous rechargeable lithium-ion batteries.

In terms of DIW, Wang et al. developed a LiFePO_4_ (LFP) composite ink containing acetylene black and multi-walled carbon nanotubes [143]. The research group successfully fabricated thick electrodes with three-dimensional patterned structures, achieving an area capacity of up to 7.5 mAThanh cm^−2^ at a thickness of 1500 μm, and demonstrating excellent cycle stability and high power density. This study indicated that 3D printing technology can effectively mitigate the problem of long ion transport paths in traditional thick electrodes, thereby significantly improving the overall performance of lithium-ion batteries.

### 3.2. Sodium-Ion Batteries (SIBs)

Compared to lithium resources, sodium (2.3 wt.%) has abundant reserves that are widely distributed and relatively easy to extract. Therefore, developing SIBs with similar chemical properties to LIBs has become an important issue nowadays. They have a similar structure to LIBs, consisting of anode materials, membranes, cathode materials, and electrolytes. Three-dimensional printing offers new design freedom and structural optimization potential for preparing electrode materials for SIBs, particularly demonstrating unique superiority in constructing electrodes with high loading, high specific capacity, and complex geometries. By precisely controlling the microstructure, 3D printing can effectively enhance the transmission of ions and electrons, overcoming problems such as long ion diffusion paths and poor conductivity in traditional thick electrodes. Carbon-based materials, due to their excellent electrical conductivity, chemical stability, and controllable porous structure, have become an ideal choice for 3D-printed electrodes.

In recent years, researchers have successfully fabricated various carbon-based electrodes using different types of 3D printing technologies (especially DIW and SLA), significantly enhancing the area capacity, rate performance and cycle stability of SIBs, and promoting the application of SIBs in high energy density and low-cost energy storage systems [144,145]. For instance, Ji et al. prepared an ink by mixing Na_3_V_2_(PO_4_)_3_ (NVP) with acetylene black (AB) and poly (vinyl difluoride), then printing electrodes via DIW, which was further assembled into a full-cell SIB [146]. The battery manifested a capacity of 21 mAh g^−1^ at a current density of 1 C, while 15 mAh g^−1^ at 10 C, and the capacity retention remained 55% after 4000 cycles. Katsuyama et al. used a commercial photocurable resin to construct a freestanding carbon lattice electrode material by means of the SLA technique, which was further assembled into a coin-cell SIB [147]. The device displayed a high areal capacity of 21.3 mAh g^−1^ at a loading of 98 mg cm^−2^, significantly surpassing that of traditional monolithic electrodes. The research will promote the development of SIB anode materials and help deepen the understanding of the ion insertion mechanism in hard carbon, thereby broadening the application of 3D-printed carbon structures.

In addition, 3D printing technologies also support customized electrode shapes and modular integration, providing new solutions for the power systems of flexible microelectronic devices. Ma et al. fabricated a 3D printing flexible sodium-ion microbattery via the DIW technique, of which the cathode consisted of Na_3_V_2_(PO_4_)_2_O_2_F (NVPF), exfoliated graphene, and carbon nanotubes. At the same time, the anode comprised carbon-coated NaTi_2_(PO_4_)_3_ (NTP), exfoliated graphene, and carbon nanotubes (Figure 7d–f) [141]. The 3D-printed sodium-ion microbattery realized a remarkable capacity of 4.5 mAh cm^−2^ at a current density of 2 mA cm^−2^, exceeding most of the reported microbatteries (Figure 7g), and the capacity retention remained 80% after 6000 cycles (Figure 7i). Notably, since the DIW ink contained carboxymethyl cellulose as a binder, the 3D-printed microbattery exhibited good flexibility, retaining a capacity of 93% of the original after being bent by 180 degrees (Figure 7h).

### 3.3. Metal–Air Batteries (MABs)

Metal–air batteries consist of a metal anode, an electrolyte, and a porous cathode (air electrode), which in turn includes a gas diffusion layer, a current collector layer, and a catalyst layer. MABs typically select different types of electrolytes based on the chemical properties of the metal, which can be classified into aqueous metal–air batteries (Zn-air battery, Mg-air battery, Fe-air battery, etc.) and non-aqueous metal–air batteries (Li-air battery, Na-air battery, K-air battery, etc.). Three-dimensional printing enables the precise control of the structure and morphology of electrodes. By designing vertical through large-pore channels, it promotes oxygen diffusion, and the porous structure provides electrolyte infiltration and storage space for active materials, thereby significantly enhancing the capacity, rate performance, and cycle stability of MABs [148,149]. Three-dimensional printed carbon-based materials show excellent performance while serving as catalyst carriers or self-supporting electrodes in MABs.

In terms of the aqueous metal–air batteries, Zhang et al. utilized DIW 3D printing technology to print a Zn-anode (Zn/carbon black/carbon nanofiber composites) and a self-supporting air cathode (GO/CNT/MnO_2_ composites) (Figure 7j) [142]. The cathode was subjected to freeze-drying and heat treatment to form a rGO/CNT/MnO_2_ porous structure. Then it was assembled into a Zn-air battery, exhibiting a discharge capacity of 670 mAh g^−1^ at a current density of 5 mA cm^−2^, with a maximum power density of 205 mW cm^−2^. The DIW-prepared Zn-battery demonstrated excellent rate performance and cycle life, capable of undergoing stable cycling for over 350 rounds (Figure 7k). Nagy et al. fabricated a polypropylene skeleton cell for a Zn-air rechargeable battery by virtue of FDM 3D printing [150]. This battery behaved decent eco-friendly properties and manifested a constant coulombic efficiency of 100% after 1000 cycles at a current density of 2 mA cm^−2^ and maintained a constant coulombic efficiency of 100%, without dendrite formation on the zinc electrode.

Regarding non-aqueous metal–air batteries, Lin et al. employed DIW 3D printing technology, utilizing a high-concentration graphene oxide ink to print self-supporting air electrodes with hierarchical porous structures [151]. After freeze-drying and thermal reduction, the obtained rGO electrodes processed controllable macro pores (350–520 μm) and micropores, and were assembled into a sodium-oxygen (Na-O_2_) battery. This 3D-printed electrode delivered a high specific capacity of 13,484.6 mAh g^−1^ at a current density of 0.2 A g^−1^, and exhibited stable cycling for over 120 cycles at 0.5 A g^−1^ (with a limited capacity of 500 mAh g^−1^), demonstrating excellent oxygen transport ability and electronic conductivity.

### 3.4. Redox Flow Batteries (RFBs)

Redox flow batteries achieve charging and discharging through the redox reactions of the active electrolyte molecules in the solution. The active electrolyte solution is stored in a storage tank, which is independent of the cell and connected to it through pipelines. The electrolyte is circulated to the cell by a pump, and an ion membrane is used to separate the positive and negative electrodes, allowing the active electrolyte molecules to undergo redox reactions in the cell, thereby achieving charge–discharge.

3D printing technologies offer unprecedented flexibility and precision for designing and manufacturing electrodes in flow batteries, particularly vanadium flow batteries (VRFB) [152,153,154]. They can enable the construction of carbon-based electrodes with customized porosity, complex flow channels, and functional modification, thereby significantly improving the mass transfer efficiency, conductivity, and electrochemical performance of the RFBs. By regulating the printing path, material composition, and post-processing techniques (such as carbonization and reduction), 3D printing can enable the optimization of both macro and micro structures of the electrodes, reduce polarization losses, and improve energy efficiency and cycle stability. Moreover, 3D printing can also enable rapid manufacturing and multi-material integration, thereby providing new approaches for electrode design and flow field optimization in RFBs.

For instance, Li et al. combined graphene oxide with Super-P (a conductive carbon black) to develop a composite ink for DIW 3D printing, and printed a rGO/Super-P aerogel composite electrode with a hierarchical porous structure, resulting in a high specific surface area of 133.15 m^2^ g^−1^ [154]. The printed electrodes were then assembled into a VRFB cell, exhibiting an excellent discharge capacity of 848.4 mAh at a current density of 80 mA cm^−2^, surpassing 14.9% of that of the traditional graphite felt electrode, and they demonstrated excellent cycling stability (capacity retention rate of 74.29% after 100 cycles). Heijden et al. utilized SLA 3D printing technology to print a model grid structure using a commercial acrylate-based resin (High Temp V2), which was subsequently carbonized under high-temperature conditions and then tested as RFB electrode materials [155]. This work systematically studied the influence of printing direction, pillar shape, and flow field design on mass transfer performance, indicating that appropriate printing parameters (including diagonal printing direction, helical pillars, and finger-like flow field) can significantly enhance the mass transfer coefficient and current density. The printed electrodes presented extraordinary mass transfer properties in non-aqueous electrolyte solutions, providing a design strategy for optimizing structural electrodes.

### 3.5. Supercapacitors (SCs)

Supercapacitors are high-performance electrochemical energy storage devices that store energy through the adsorption/desorption process of ions at the electrode, electrolyte surface, or near-surface. Due to their unique advantages (high output power, fast response, long lifespan, etc.), supercapacitors play an important role in energy storage, transportation, industry, and other fields [156,157]. Three-dimensional printing technology provides highly customizable design freedom for the preparation of SCs electrodes, enabling the layer-by-layer deposition of carbon-based materials (including activated carbon, graphene, graphene oxide, and their composite materials) and the construction of complex three-dimensional porous structures, significantly improving the quality of loading, ion transfer efficiency, and electrochemical performance of the electrodes. Additionally, 3D printing can also achieve the integrated molding of all-solid-state devices, including the simultaneous printing of electrodes and electrolytes, avoiding the complexity of traditional multi-step processes and promoting the development of high-performance, flexible, and miniaturized supercapacitors.

**Figure 8:**
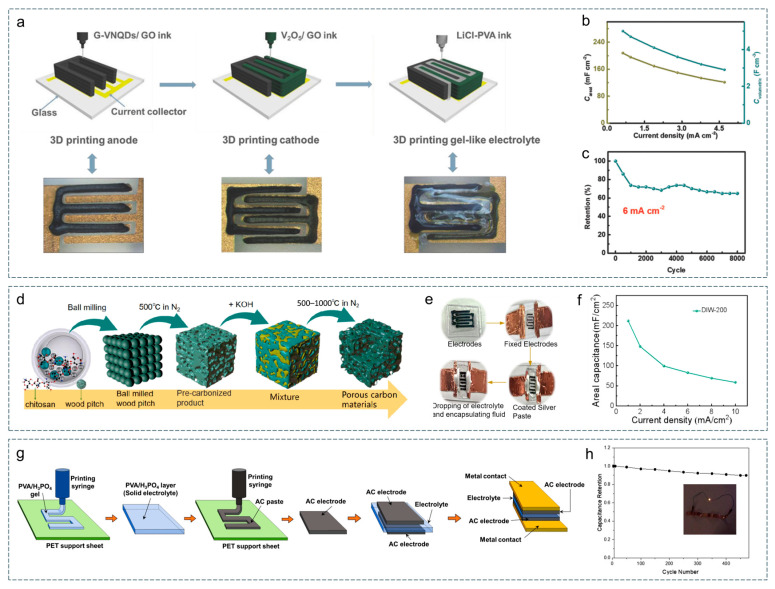
(**a**) Schematic of 3D printing an asymmetric micro-supercapacitor. (**b**) Areal and volumetric capacitances, and (**c**) cycling performance of the printed device, ref. [158] copyright 2018, Wiley-VCH. (**d**) Illustration of the preparation process for N-doped porous carbon materials from wood pitch. (**e**) Fabrication process of the micro-supercapacitor, and (**f**) surface capacitance of the printed electrodes, ref. [159] copyright 2025, Elsevier. (**g**) Schematic of the SC fabrication, and (**h**) cycling performance (*inset*: LED powered by two SCs connected in series), ref. [160] copyright 2020, Elsevier.

Shen et al. used V_2_O_5_/Graphene oxide (GO) composites as anode ink and graphene-vanadium nitride quantum dots (G-VNQDs)/GO composites as cathode ink to construct an asymmetric micro-supercapacitor by means of DIW 3D printing technology (Figure 8a). The printed SCs exhibited excellent electrochemical properties, demonstrating an areal capacitance of 207.9 mF cm^−2^ at an electrochemical potential window of 1.6 V (Figure 8b). In addition, the SCs also showed good cycle stability, with a capacity retaining 65% after 8000 cycles (Figure 8c) [158].

Additionally, the 3D-printed carbon-based electrochemical energy storage devices can be fabricated from an extraordinarily eco-friendly resource. For instance, Weng et al. prepared a nitrogen-doped porous carbon material using wood pitch as precursor material, which was further combined with PEDOT:PSS and cellulose nanocrystals to develop a conductive ink (Figure 8d) [159]. By means of DIW 3D printing, the researcher constructed a micro-interdigital electrode (a typical kind of SCs) (Figure 8e). The printed electrodes exhibited an ultra-high specific surface area of 3457.2 m^2^ g^−1^ and excellent electrochemical properties, which manifested an areal specific capacitance of 213 mF cm^−2^ at a current density of 1 mA cm^−2^ (Figure 8f), an energy density of 29.42 μWh cm^−2^, and a power density of 1.72 mW cm^−2^. Meanwhile, the printed SCs exhibited an extraordinary rate performance and cycling stability, with the capacity retention remaining 91.5% after 5000 cycles. Idrees et al. utilized a package waste-derived porous carbon as an ink precursor to print a supercapacitor via DIW 3D printing (Figure 8g) [160]. The printed SC demonstrated a high areal capacitance of 328.95 mF cm^2^ and a specific capacitance of 3.48 F g^−1^ at 2.5 mA. And the device also processed good cycling stability, maintaining a capacity retention of 90% after 500 cycles (Figure 8h).

In addition, 3D microsupercapacitors (MSC) and Li-ion capacitors are also noticeable carbon-based printed energy-storage devices. For instance, Wang et al. utilized bipolar electrochemical methods to deposit vertically aligned reduced graphene oxide on a gold finger micro-current collector, thereby fabricating a high-frequency responsive MSC [161]. The printed device demonstrated an average specific capacitance of 640 μF cm^−2^ at a scanning rate of 2 mV s^−1^ and maintained excellent stability after 50,000 cycles. This MSC exhibits comparable filtering performance to commercial aluminum electrolytic capacitors in an AC filter circuit, with a smaller volume, and is suitable for high-frequency microelectronic system integration. This team also developed an on-chip lithium-ion capacitor based on carbon micro-electromechanical system (C-MEMS) [162]. They used a 3D finger-type carbon microelectrode array as the capacitive negative electrode, and LiFePO_4_ as the battery-type positive electrode. The surface area and surface functional groups of the carbon electrode are enhanced by oxygen plasma treatment to improve its capacitive performance. This device uses a 1 M Li_2_SO_2_ aqueous electrolyte and operates within a voltage window of 0–1.4 V, achieving an area energy density of up to 5.03 μWh cm^−2^, which is approximately 5 times that of symmetrical carbon-based electrochemical capacitors. The study demonstrates the potential of C-MEMS technology in asymmetric energy storage devices and provides a new path for high-performance, integrable microelectronic energy storage systems.

In general, 3D printing technology, with its unique advantage of precisely designing and controllably constructing material structures in three-dimensional space, provides unprecedented preparation freedom for high-performance electrochemical energy storage devices. In various energy storage systems such as LIBs, SIBs, MABs, RFBs, and SCs, 3D-printed carbon-based materials have achieved the controllable construction of hierarchical pore structures, optimized the ion and electron transport paths, and significantly increased the specific surface area and active substance loading of the electrodes, thereby effectively enhancing the energy density, power density, and cycle stability of the devices. Three-dimensional printing proposes feasible strategies for developing the next-generation high-performance, flexible, and miniaturized electrochemical energy storage devices. Here we list a performance comparison of the various printed devices in Table 2.

**Table 2:** Comparison of performances of different printed electrochemical energy storage devices.

Device	Carbon Material	3D Printing Methods	Capacitance	Energy Density/Power Density	Stability (Cycle Number)	Ref.
LIBs	LFP/AB/CNT	DIW	7.5 mA h cm^−2^	69.41 J cm^−2^ /2.99 mW cm^−2^	51.8% (115)	[143]
	CB-doped PPy, Graphene	FDM	345 mAh g^−1^ at 20 mA g^−1^	\	96% (350)	[163]
	Carbon filament	FDM	69.1 mAh g^−1^ at 0.036 mA cm^−2^	\	84.4% (200)	[140]
	Carbon-coated LFP	IJP	80 mAh g^−1^	\	no loss at 9 C (100)	[164]
SIBs	Carbon microlattice	SLA	21.3 mAh cm^−2^ at 98 mg cm^−2^	\	/	[147]
	NVP/AB/PVDF	DIW	21 mAh g^−1^ at 1 C	\	55% (4000)	[146]
	CNT/Graphene/NTP, NVPF	DIW	4.5 mAh cm^−2^ at 2 mA cm^−2^	7.33 mAh cm^−2^/	80% (6000)	[141]
MABs	GO/CNT/MnO_2_	DIW	670 mAh g^−1^ at 5 mA cm^−2^	/205 mW cm^−2^	stable cycling over 350	[142]
	rGO	DIW	13,484.6 mAh g^−1^ at 0.2 A g^−1^	\	stable cycling over 120	[151]
	CNT	DIW	110 mAh g^−1^ at 50 mA g^−1^	\	81% (30)	[148]
RFBs	rGO/Super-P (CB)	DIW	848.4 mAh	\	74.29% (100)	[154]
	Graphene Aerogel	DIW	714.9 mAh	\	95% (100)	[152]
SCs	V_2_O_5_/GO, (G-VNQDs)/GO	DIW	207.9 mF cm^−2^ at 1.6 V	73.9 μWh cm^−2^ /3.77 mW/cm^−2^	65% (8000)	[158]
	N-doped porous carbon	DIW	213 mF cm^−2^ at 1 mA cm^−2^	29.42 μWh cm^−2^ /1.72 mW cm^−2^	91.5% (5000)	[159]
	Package waste-derived porous carbon	DIW	328.95 mF cm^2^	0.484 Wh kg^−1^ /15.01 W kg^−1^	90% (500)	[160]
	Graphene/PEDOT:PSS	IJP	13.8 mF cm^−2^ at 10 mV s^−1^	~1 μWh cm^−2^ /~10 mW/cm^−2^	80% (2000)	[165]
	N-doped sucrose-derived carbon	IJP	151 F cm^−2^ at 3.9 mF cm^−2^	0.9 mWh cm^−3^ /0.4 W/cm^−3^	96% (10,000)	[166]
	rGO-CNT-PEDOT:PSS	AJP	21.7 F g^−1^ at 0.5 A g^−1^	\	88% (10,000)	[167]

For the above-mentioned 3D-printed devices, there is an issue that needs to be considered, which is the degradation that occurs during their long-term cycling process (such as mechanical delamination, electrolyte drying, and binder decomposition). Mechanical delamination refers to the separation of printed layers in printed electrodes during long-term cycles. This directly undermines the structural integrity of the electrode and the pre-designed ion/electron transport channels. This is mainly because of the inherent weakness of the interlayer bonding force. We believe that this problem can be addressed by optimizing ink performance, printing parameters, and rationally designing the device structure (such as a hierarchical porous structure). Three-dimensional printing can fabricate precise structures with a high specific surface area to enhance electrochemical properties. However, this also means that the interface area between the electrode and the electrolyte is huge, providing more channels for solvent evaporation. During long-term cycling, especially at higher temperatures, the solvent components in the electrolyte may slowly evaporate, leading to a decrease in ionic conductivity and deterioration of the electrode/electrolyte contact interface. In addition, due to the limited electrochemical stability window of the binder material, oxidation and decomposition may occur at high voltages. Therefore, this poses new requirements for the optimization of ink material preparation, printing process, and post-treatment process in the future.

## 4. Perspectives and Outlook

As noted above, 3D printing technology provides a revolutionary solution for designing and manufacturing carbon-based electrochemical energy storage devices. This review systematically summarizes the material properties and performance advantages of different carbon-based materials (including Graphene, CNTs, AC, carbon fibers, and their composite materials with metal oxides, polymers, and biomass derivatives), and the key factors in the design of carbon-based inks. It compares the technical characteristics of typical 3D printing technologies (including DIW, IJP, AJP, FDM, and SLA) and their applications in electrochemical energy storage devices (including LIBs/SIBs, MABs, RFBs, and SCs). Through precise control of the structure and microscopic morphology of the electrodes, the synergistic optimization of ion transport paths, electronic conduction networks, and mechanical stability is achieved, significantly enhancing the energy density, power density, and cycle life of the devices.

Despite the rapid development and broad application of 3D printing technologies in electrochemical energy storage devices, many dispiriting challenges remain to be addressed. This is manifested in the following aspects.

(1) **Further Design of Carbon-Based Ink Materials**. Ink design is the core and prerequisite for developing high-performance 3D-printed carbon-based electrochemical energy storage devices. Therefore, the research focus in the future remains on the design of the ink and its functional materials. For instance, developing low-concentration, electrochemically stable additives is a significant direction. Currently, carbon-based ink, especially for DIW, often relies on high-concentration viscosity regulators. These additives are usually non-conductive, thereby affecting device performance. In the future, low-concentration, highly stable additives should be developed to maintain printing performance while enhancing energy density and cycle life. This paper systematically elaborates on the fabrication of carbon-based inks, from material selection and formulation design to performance optimization. The core lies in achieving a balance among printability, stability, and functionality. In addition, considering environmental protection, future efforts should be made to develop new types of green solvent systems for carbon-based 3D-printing inks with low toxicity and good biocompatibility.
(2) **Printing accuracy and process optimization**. Improving printing accuracy and optimizing printing processes are among the core challenges in advancing 3D-printed electrochemical energy storage devices from the laboratory to commercial applications. To achieve this goal, here we provide two reference suggestions. Firstly, develop nozzles with diameters of micrometers or even sub-micrometers (for example, reducing the DIW nozzle from the conventional tens of micrometers to a range of 1–5 μm), and simultaneously develop new functional inks with excellent shear thinning behavior and high yield stress, thereby stabilizing the print line width of DIW at less than 10 μm and achieving an increase in the feature size of FDM printing from the current 100 μm scale to 50 μm or higher precision. Secondly, optimize the equipment system. For instance, by optimizing the optical path system, developing new photosensitive resins, and adopting gray-scale exposure strategies, the inherent high resolution advantage of SLA (~10–100 μm) can be further extended to the micro-nano scale (<10 μm), and efforts are made to develop innovative printing algorithms and designs without support structures or water-soluble/tearable supports, to significantly reduce post-processing steps and protect the integrity of fine structures.
(3) **Multi-material Collaborative Optimization**. Three-dimensional printing technology is primarily focused on printing single materials or simple composite materials. To achieve more high-performance and multi-functional energy storage devices, exploring multi-material integration systems is a feasible solution. Studying the interfacial interactions between different materials and optimizing the distribution and connection methods of materials to achieve collaborative improvement in performance. For example, in lithium-ion batteries, by 3D printing to design gradient structure electrodes, the utilization rate of active materials can be maximized while reducing the problem of stress concentration.
(4) **Integrated Fabrication of Electrochemical Energy Storage Devices**. By utilizing 3D printing technology to simultaneously print electrodes, electrolytes, and current collectors, the overall manufacturing process of energy storage devices can be achieved, which can significantly reduce interface contact resistance in traditional manufacturing processes and improve the energy density and power density of the devices. Research on multi-material integration systems needs to overcome technical challenges, including material compatibility, deformation control during the printing process, and interface bonding strength. In the future, the development of new composite ink systems and high-precision multi-nozzle printing technologies can be used to promote this direction.
(5) **4D Printing and Dynamic Control of the Electrochemical Energy Storage Devices**. Four-dimensional printing is an extension of 3D printing, which can endow the printing materials with dynamic response capabilities in the time dimension, such as shape memory, self-repairing, and environmental responsiveness. For instance, when the energy storage device is subjected to external mechanical deformation (such as bending or stretching), the 4D-printed carbon-based material can return to its original shape, thereby ensuring the stability and performance of the device. By introducing microcapsulated self-repairing agents or dynamic chemical bonds (such as hydrogen bonds, metal coordination bonds) into the carbon-based material, the 4D-printed electrode can automatically repair local damage and extend the service life of the device. Additionally, when combined with intelligent materials (such as thermosensitive, hygroscopic, and pH-sensitive materials), 4D-printed carbon-based materials can achieve a dynamic response to environmental conditions, thereby optimizing the operating state of the energy storage device. Under different temperature conditions, the material can automatically adjust the pore structure to optimize the ion transport efficiency.
(6) **Balance the Trade-off between Mechanical Stability and Electrochemical Properties of Printed Devices**. For 3D-printed carbon-based energy storage devices, there is an inherent contradiction between mechanical properties and electrochemical properties. Although a high-porosity structure is beneficial for ion transport and active substance loading, which can enhance energy density and rate performance, but it may sacrifice mechanical strength, leading to easy cracking of the device. Adding polymers (such as PLA) to enhance flexibility can improve toughness, but usually significantly reduces conductivity. Here we presented two proposals. Through composite material design and structural optimization, this contradiction can be balanced to some extent. For example, using rGO and CNTs composite ink can maintain the conductive network while buffering stress through an elastic interface. Constructing biomimetic porous or stretchable configurations (such as wave-shaped electrodes) can balance ion migration and deformation adaptability. In the future, by combining multi-material printing, nanoscale structure control, and intelligent post-processing techniques, it is expected to achieve a synergistic improvement in mechanical stability and electrochemical performance at the microscopic level.
(7) **Large-scale Manufacturing of Printed Devices and the Establishment of a Standardized Evaluation System**. 3D-printed carbon-based electrochemical energy storage devices are currently at a critical stage of transitioning from the laboratory to industrial applications. This is a comprehensive issue involving materials, processes, quality control, and industrial chain collaboration. In terms of material and formulation, the optimized formulas developed in the laboratory often experience performance degradation and batch-to-batch instability when scaled up. At the process level, the precise dispersion, purification, and printing conditions in the laboratory are difficult to perfectly replicate on cost-effective industrial production lines. Moreover, the absence of quality control and standardization systems makes it impossible to ensure the consistency and reliability of the products, significantly increasing the application risks and concerns of downstream electronic manufacturers, thereby inhibiting market adoption. From a cost and industrial chain perspective, the high cost of high-performance carbon materials and their reliance on expensive raw materials/processes make the final products lack competitiveness in terms of economy. Therefore, breaking through these limitations requires not only innovation in materials but also breakthroughs in process engineering, printing equipment, and close collaboration of the entire industrial ecosystem. Additionally, future efforts should focus on research aimed at reducing environmental impact and manufacturing costs. At the same time, establishing performance testing and evaluation standards applicable to 3D-printed electrodes is necessary to promote the standardization and commercialization of the technology.

By enabling multi-scale structural innovation, 3D printing technologies maximize the inherent potential of carbon-based materials. Furthermore, through advances in carbon material design, ink formulation, printing technologies, and devices, coupled with cross-disciplinary integration and intelligent manufacturing, 3D printing holds great promise for accelerating the development of next-generation electrochemical energy storage devices. Such devices are expected to be high-performance, flexible, and customizable, providing critical technical support for global energy transition and the achievement of carbon neutrality goals.

## Figures and Tables

**Table 1:** Comparison of intrinsic properties of different elemental carbon materials.

Material	Specific Surface Area (m^2^ g^−1^)	Conductivity (S g^−1^)	Young’s Modulus (TPa)	Structural Features
**Graphene**	~2630	~10^6^	~1	Two-dimensional lamellar layer
**CNTs**	200~800	~10^5^	~1	One-dimensional tubular
**AC**	500~2200	~10^2^	Low	Multi-level porous
**Carbon Fiber**	<10	10^2^~10^4^	0.1~0.9	Fibrous and weavable

## Data Availability

The original contributions presented in this study are included in the article. Further inquiries can be directed to the corresponding authors.

## References

[1] Dunn B., Kamath H., Tarascon J.-M. (2011). Electrical energy storage for the grid a battery of choices. Science.

[2] Larcher D., Tarascon J.M. (2014). Towards greener and more sustainable batteries for electrical energy storage. Nat. Chem..

[3] Goodenough J.B., Park K.-S. (2013). The Li-Ion Rechargeable Battery: A Perspective. J. Am. Chem. Soc..

[4] Yang Y., Liu X., Zhu Z., Zhong Y., Bando Y., Golberg D., Yao J., Wang X. (2018). The Role of Geometric Sites in 2D Materials for Energy Storage. Joule.

[5] Landi B.J., Ganter M.J., Cress C.D., DiLeo R.A., Raffaelle R.P. (2009). Carbon nanotubes for lithium ion batteries. Energy Environ. Sci..

[6] Jin T., Han Q., Jiao L. (2019). Binder-Free Electrodes for Advanced Sodium-Ion Batteries. Adv. Mater..

[7] Liu N., Gao Y. (2017). Recent Progress in Micro-Supercapacitors with In-Plane Interdigital Electrode Architecture. Small.

[8] Edgar J., Tint S. (2015). Additive manufacturing technologies: 3D printing, rapid prototyping, and direct digital manufacturing. Johns. Matthey Technol. Rev..

[9] Tian X., Jin J., Yuan S., Chua C.K., Tor S.B., Zhou K. (2017). Emerging 3D-Printed Electrochemical Energy Storage Devices: A Critical Review. Adv. Energy Mater..

[10] Egorov V., Gulzar U., Zhang Y., Breen S., O’Dwyer C. (2020). Evolution of 3D Printing Methods and Materials for Electrochemical Energy Storage. Adv. Mater..

[11] Lacey S.D., Kirsch D.J., Li Y., Morgenstern J.T., Zarket B.C., Yao Y., Dai J., Garcia L.Q., Liu B., Gao T. (2018). Extrusion-Based 3D Printing of Hierarchically Porous Advanced Battery Electrodes. Adv. Mater..

[12] Vernardou D., Vasilopoulos K.C., Kenanakis G. (2017). 3D printed graphene-based electrodes with high electrochemical performance. Appl. Phys. A.

[13] Zhu C., Liu T., Qian F., Chen W., Chandrasekaran S., Yao B., Song Y., Duoss E.B., Kuntz J.D., Spadaccini C.M. (2017). 3D printed functional nanomaterials for electrochemical energy storage. Nano Today.

[14] Kim C., Ahn B.Y., Cho S.H., Jung J.W., Kim I.D. (2025). 3D Printing for Energy Storage Devices: Advances, Challenges, and Future Directions. Adv. Mater..

[15] Zhang F., Wei M., Viswanathan V.V., Swart B., Shao Y., Wu G., Zhou C. (2017). 3D printing technologies for electrochemical energy storage. Nano Energy.

[16] Zhai Y., Dou Y., Zhao D., Fulvio P.F., Mayes R.T., Dai S. (2011). Carbon Materials for Chemical Capacitive Energy Storage. Adv. Mater..

[17] Xu Y., Lin Z., Zhong X., Huang X., Weiss N.O., Huang Y., Duan X. (2014). Holey graphene frameworks for highly efficient capacitive energy storage. Nat. Commun..

[18] Sun H., Mei L., Liang J., Zhao Z., Lee C., Fei H., Ding M., Lau J., Li M., Wang C. (2017). Three-dimensional holey-grapheneniobia composite architectures for ultrahigh-rate energy storage. Science.

[19] Zhou F., Han S., Qian Q., Zhu Y. (2019). 3D printing of free-standing and flexible nitrogen doped graphene/polyaniline electrode for electrochemical energy storage. Chem. Phys. Lett..

[20] Sang Tran T., Dutta N.K., Roy Choudhury N. (2019). Graphene-Based Inks for Printing of Planar Micro-Supercapacitors: A Review. Materials.

[21] Yu W., Zhou H., Li B.Q., Ding S. (2017). 3D Printing of Carbon Nanotubes-Based Microsupercapacitors. ACS Appl. Mater. Interfaces.

[22] Jiang Y., Guo F., Liu Y., Xu Z., Gao C. (2021). Three-dimensional printing of graphene-based materials for energy storage and conversion. SusMat.

[23] Foster C.W., Down M.P., Zhang Y., Ji X., Rowley-Neale S.J., Smith G.C., Kelly P.J., Banks C.E. (2017). 3D Printed Graphene Based Energy Storage Devices. Sci. Rep..

[24] Lyu Z., Lim G.J.H., Koh J.J., Li Y., Ma Y., Ding J., Wang J., Hu Z., Wang J., Chen W. (2021). Design and Manufacture of 3D-Printed Batteries. Joule.

[25] Yang H., Fang L., Yuan Z., Teng X., Qin H., He Z., Wan Y., Wu X., Zhang Y., Guan L. (2023). Machine learning guided 3D printing of carbon microlattices with customized performance for supercapacitive energy storage. Carbon.

[26] Wang J., Shi Z., Gong J., Zhou X., Li J., Lyu Z. (2024). 3D printing of graphene-based aerogels and their applications. FlatChem.

[27] Park S., Cao Z., Sung D.H., Fu K.K. (2023). High-Loaded Electrode Filaments for Additive Manufacturing of Structural Batteries. Adv. Energy Mater..

[28] Zhu C., Han T.Y.-J., Duoss E.B., Golobic A.M., Kuntz J.D., Spadaccini C.M., Worsley M.A. (2015). Highly compressible 3D periodic graphene aerogel microlattices. Nat. Commun..

[29] Sun C., Liu S., Shi X., Lai C., Liang J., Chen Y. (2020). 3D printing nanocomposite gel-based thick electrode enabling both high areal capacity and rate performance for lithium-ion battery. Chem. Eng. J..

[30] Ma J., Zheng S., Fu Y., Wang X., Qin J., Wu Z.-S. (2024). The status and challenging perspectives of 3D-printed micro-batteries. Chem. Sci..

[31] Zhang Y., Tan Y.-W., Stormer H.L., Kim P. (2005). Experimental observation of the quantum Hall effect and Berry’s phase in graphene. Nature.

[32] You R., Liu Y.Q., Hao Y.L., Han D.D., Zhang Y.L., You Z. (2019). Laser Fabrication of Graphene-Based Flexible Electronics. Adv. Mater..

[33] Wang H., Cui L.-F., Yang Y., Casalongue H.S., Robinson J.T., Liang Y., Cui Y., Dai H. (2010). Mn_3_O_4_-Graphene Hybrid as a High-Capacity Anode Material for Lithium Ion Batteries. J. Am. Chem. Soc..

[34] Mayorov A.S., Gorbachev R.V., Morozov S.V., Britnell L., Jalil R., Ponomarenko L.A., Blake P., Novoselov K.S., Watanabe K., Taniguchi T. (2011). Micrometer-Scale Ballistic Transport in Encapsulated Graphene at Room Temperature. Nano Lett..

[35] Huang X., Qi X., Boey F., Zhang H. (2012). Graphene-based composites. Chem. Soc. Rev..

[36] Balandin A.A. (2011). Thermal properties of graphene and nanostructured carbon materials. Nat. Mater..

[37] Lee C., Wei X., Kysar J.W., Hone J. (2008). Measurement of the Elastic Properties and Intrinsic Strength of Monolayer Graphene. Science.

[38] Huang X., Yin Z., Wu S., Qi X., He Q., Zhang Q., Yan Q., Boey F., Zhang H. (2011). Graphene-Based Materials: Synthesis, Characterization, Properties, and Applications. Small.

[39] Li D., Müller M.B., Gilje S., Kaner R.B., Wallace G.G. (2008). Processable aqueous dispersions of graphene nanosheets. Nat. Nanotechnol..

[40] Ramesha G.K., Sampath S. (2009). Electrochemical Reduction of Oriented Graphene Oxide Films: An in Situ Raman Spectroelectrochemical Study. J. Phys. Chem. C Lett..

[41] Wei M., Zhang F., Wang W., Alexandridis P., Zhou C., Wu G. (2017). 3D direct writing fabrication of electrodes for electrochemical storage devices. J. Power Sources.

[42] Ishikawa F.N., Chang H.-k., Ryu K., Chen P.-c., Badmaev A., Arco L.G.D., Shen G., Zhou C. (2009). Transparent Electronics Based on Transfer Printed Aligned Carbon Nanotubes on Rigid and Flexible Substrates. ACS Nano.

[43] Yu W., Li B.Q., Ding S.J. (2016). Electroless fabrication and supercapacitor performance of CNT@NiO-nanosheet composite nanotubes. Nanotechnology.

[44] Sun G., An J., Chua C.K., Pang H., Zhang J., Chen P. (2015). Layer-by-layer printing of laminated graphene-based interdigitated microelectrodes for flexible planar micro-supercapacitors. Electrochem. Commun..

[45] El-Kady M.F., Kaner R.B. (2013). Scalable fabrication of high-power graphene micro-supercapacitors for flexible and on-chip energy storage. Nat. Commun..

[46] Steldinger H., Esposito A., Brunnengräber K., Gläsel J., Etzold B.J.M. (2019). Activated Carbon in the Third Dimension—3D Printing of a Tuned Porous Carbon. Adv. Sci..

[47] Yeong W.Y., Goh G.D. (2020). 3D Printing of Carbon Fiber Composite: The Future of Composite Industry?. Matter.

[48] Sanei S.H.R., Popescu D. (2020). 3D-Printed Carbon Fiber Reinforced Polymer Composites: A Systematic Review. J. Compos. Sci..

[49] Parandoush P., Zhou C., Lin D. (2018). 3D Printing of Ultrahigh Strength Continuous Carbon Fiber Composites. Adv. Eng. Mater..

[50] Blyweert P., Nicolas V., Fierro V., Celzard A. (2021). 3D printing of carbon-based materials: A review. Carbon.

[51] Jost K., Stenger D., Perez C.R., McDonough J.K., Lian K., Gogotsi Y., Dion G. (2013). Knitted and screen printed carbon-fiber supercapacitors for applications in wearable electronics. Energy Environ. Sci..

[52] Wang H., Zhang C., Chen Z., Liu H.K., Guo Z. (2015). Large-scale synthesis of ordered mesoporous carbon fiber and its application as cathode material for lithium–sulfur batteries. Carbon.

[53] Sun M., Shi Z., Han Q. (2025). Structure-function integrated carbon fiber reinforced composites with enhanced mechanical robustness and electrochemical stability. Chem. Eng. J..

[54] Yue T., Shen B., Gao P. (2022). Carbon material/MnO_2_ as conductive skeleton for supercapacitor electrode material: A review. Renew. Sustain. Energy Rev..

[55] Vangari M., Pryor T., Jiang L. (2013). Supercapacitors: Review of Materials and Fabrication Methods. J. Energy Eng..

[56] Choudhary N., Li C., Moore J., Nagaiah N., Zhai L., Jung Y., Thomas J. (2017). Asymmetric Supercapacitor Electrodes and Devices. Adv. Mater..

[57] Hui N., Chai F., Lin P., Song Z., Sun X., Li Y., Niu S., Luo X. (2016). Electrodeposited Conducting Polyaniline Nanowire Arrays Aligned on Carbon Nanotubes Network for High Performance Supercapacitors and Sensors. Electrochim. Acta.

[58] Yan J., Wang Q., Wei T., Fan Z. (2013). Recent Advances in Design and Fabrication of Electrochemical Supercapacitors with High Energy Densities. Adv. Energy Mater..

[59] Choi J.R., Lee J.W., Yang G., Heo Y.-J., Park S.-J. (2020). Activated Carbon/MnO_2_ Composites as Electrode for High Performance Supercapacitors. Catalysts.

[60] Dubey R., Guruviah V. (2019). Review of carbon-based electrode materials for supercapacitor energy storage. Ionics.

[61] Lei R., Zhang H., Lei W., Li D., Fang Q., Ni H., Gu H. (2019). MnO_2_ nanowires electrodeposited on freestanding graphenated carbon nanotubes as binder-free electrodes with enhanced supercapacitor performance. Mater. Lett..

[62] Jia H., Cai Y., Zheng X., Lin J., Liang H., Qi J., Cao J., Feng J., Fei W. (2018). Mesostructured Carbon Nanotube-on-MnO_2_ Nanosheet Composite for High-Performance Supercapacitors. ACS Appl. Mater. Interfaces.

[63] Xu M., Fu N., Wang X., Yang Z. (2020). A high energy density flexible symmetric supercapacitor based on Al-doped MnO_2_ nanosheets @ carbon cloth electrode materials. J. Mater. Sci. Mater. Electron..

[64] Yao B., Chandrasekaran S., Zhang J., Xiao W., Qian F., Zhu C., Duoss E.B., Spadaccini C.M., Worsley M.A., Li Y. (2019). Efficient 3D Printed Pseudocapacitive Electrodes with Ultrahigh MnO_2_ Loading. Joule.

[65] Wu D., Xie X., Zhang Y., Zhang D., Du W., Zhang X., Wang B. (2020). MnO_2_/Carbon Composites for Supercapacitor: Synthesis and Electrochemical Performance. Front. Mater..

[66] Xiong C., Li T., Zhao T., Dang A., Ji X., Li H., Etesami M. (2018). Three-Dimensional Graphene/MnO_2_ Nanowalls Hybrid for High-Efficiency Electrochemical Supercapacitors. Nano.

[67] Wang H., Fu Q., Pan C. (2019). Green mass synthesis of graphene oxide and its MnO_2_ composite for high performance supercapacitor. Electrochim. Acta.

[68] Mondal S., Rana U., Malik S. (2015). Graphene quantum dot-doped polyaniline nanofiber as high performance supercapacitor electrode materials. Chem. Commun..

[69] Sun J., Cui B., Chu F., Yun C., He M., Li L., Song Y. (2018). Printable Nanomaterials for the Fabrication of High-Performance Supercapacitors. Nanomaterials.

[70] Naguib M., Kurtoglu M., Presser V., Lu J., Niu J., Heon M., Hultman L., Gogotsi Y., Barsoum M.W. (2011). Two-Dimensional Nanocrystals Produced by Exfoliation of Ti_3_AlC_2_. Adv. Mater..

[71] Li X., Huang Z., Shuck C.E., Liang G., Gogotsi Y., Zhi C. (2022). MXene chemistry, electrochemistry and energy storage applications. Nat. Rev. Chem..

[72] Shayesteh Zeraati A., Mirkhani S.A., Sun P., Naguib M., Braun P.V., Sundararaj U. (2021). Improved synthesis of Ti_3_C_2_T_x_ MXenes resulting in exceptional electrical conductivity, high synthesis yield, and enhanced capacitance. Nanoscale.

[73] Jiang Q., Kurra N., Alhabeb M., Gogotsi Y., Alshareef H.N. (2018). All Pseudocapacitive MXene-RuO_2_ Asymmetric Supercapacitors. Adv. Energy Mater..

[74] Xu Y., Pan B., Li W.-S., Dong L., Wang X., Zhao F.-G. (2021). High-Performance Flexible Asymmetric Supercapacitor Paired with Indanthrone@Graphene Heterojunctions and MXene Electrodes. ACS Appl. Mater. Interfaces.

[75] Alhabeb M., Maleski K., Anasori B., Lelyukh P., Clark L., Sin S., Gogotsi Y. (2017). Guidelines for Synthesis and Processing of Two-Dimensional Titanium Carbide (Ti_3_C_2_T_x_ MXene). Chem. Mater..

[76] Kai W., Liwei L., Wen X., Shengzhe Z., Yong L., Hongwei Z., Zongqiang S. (2017). Electrodeposition Synthesis of PANI/MnO2/Graphene Composite Materials and its Electrochemical Performance. Int. J. Electrochem. Sci..

[77] Hosseini M.G., Shahryari E., Yardani Sefidi P. (2021). Polyaniline grafted chitosan/GO-CNT/Fe_3_O_4_ nanocomposite as a superior electrode material for supercapacitor application. J. Appl. Polym. Sci..

[78] Yoo D., Kim J., Lee S.H., Cho W., Choi H.H., Kim F.S., Kim J.H. (2015). Effects of one- and two-dimensional carbon hybridization of PEDOT:PSS on the power factor of polymer thermoelectric energy conversion devices. J. Mater. Chem. A.

[79] Liu T., Li C., Liu H., Zhang S., Yang J., Zhou J., Yu J., Ji M., Zhu C., Xu J. (2021). Tear resistant Tyvek/Ag/poly(3,4-ethylenedioxythiophene): Polystyrene sulfonate (PEDOT:PSS)/carbon nanotubes electrodes for flexible high-performance supercapacitors. Chem. Eng. J..

[80] Abshirini M., Charara M., Liu Y., Saha M., Altan M.C. (2018). 3D Printing of Highly Stretchable Strain Sensors Based on Carbon Nanotube Nanocomposites. Adv. Eng. Mater..

[81] Ziaee M., Johnson J.W., Yourdkhani M. (2022). 3D Printing of Short-Carbon-Fiber-Reinforced Thermoset Polymer Composites via Frontal Polymerization. ACS Appl. Mater. Interfaces.

[82] Ateeq M., Shafique M., Azam A., Rafiq M. (2023). A review of 3D printing of the recycled carbon fiber reinforced polymer composites: Processing, potential, and perspectives. J. Mater. Res. Technol..

[83] Zhang H., Yang D., Sheng Y. (2018). Performance-driven 3D printing of continuous curved carbon fibre reinforced polymer composites: A preliminary numerical study. Compos. Part B Eng..

[84] Celiktas M.S., Alptekin F.M. (2019). Conversion of model biomass to carbon-based material with high conductivity by using carbonization. Energy.

[85] Zhu Y.E., Yang L., Sheng J., Chen Y., Gu H., Wei J., Zhou Z. (2017). Fast Sodium Storage in TiO_2_@CNT@C Nanorods for High-Performance Na-Ion Capacitors. Adv. Energy Mater..

[86] Zhang G., Liu X., Wang L., Fu H. (2022). Recent advances of biomass derived carbon-based materials for efficient electrochemical energy devices. J. Mater. Chem. A.

[87] Zhang L., Zhang F., Yang X., Leng K., Huang Y., Chen Y. (2013). High-Performance Supercapacitor Electrode Materials Prepared from Various Pollens. Small.

[88] Wei J., Iglesia E. (2004). Isotopic and kinetic assessment of the mechanism of reactions of CH_4_ with CO_2_ or H_2_O to form synthesis gas and carbon on nickel catalysts. J. Catal..

[89] Bommier C., Xu R., Wang W., Wang X., Wen D., Lu J., Ji X. (2015). Self-activation of cellulose: A new preparation methodology for activated carbon electrodes in electrochemical capacitors. Nano Energy.

[90] Ling Z., Wang Z., Zhang M., Yu C., Wang G., Dong Y., Liu S., Wang Y., Qiu J. (2015). Sustainable Synthesis and Assembly of Biomass-Derived B/N Co-Doped Carbon Nanosheets with Ultrahigh Aspect Ratio for High-Performance Supercapacitors. Adv. Funct. Mater..

[91] Kubo S., White R.J., Yoshizawa N., Antonietti M., Titirici M.-M. (2011). Ordered Carbohydrate-Derived Porous Carbons. Chem. Mater..

[92] Hu B., Wang K., Wu L., Yu S.H., Antonietti M., Titirici M.M. (2010). Engineering Carbon Materials from the Hydrothermal Carbonization Process of Biomass. Adv. Mater..

[93] Liu X., Giordano C., Antonietti M. (2013). A Facile Molten-Salt Route to Graphene Synthesis. Small.

[94] Lv W., Wen F., Xiang J., Zhao J., Li L., Wang L., Liu Z., Tian Y. (2015). Peanut shell derived hard carbon as ultralong cycling anodes for lithium and sodium batteries. Electrochim. Acta.

[95] Jiang J., Zhu J., Ai W., Fan Z., Shen X., Zou C., Liu J., Zhang H., Yu T. (2014). Evolution of disposable bamboo chopsticks into uniform carbon fibers: A smart strategy to fabricate sustainable anodes for Li-ion batteries. Energy Environ. Sci..

[96] Husmann S., Zarbin A.J.G., Dryfe R.A.W. (2020). High-performance aqueous rechargeable potassium batteries prepared via interfacial synthesis of a Prussian blue-carbon nanotube composite. Electrochim. Acta.

[97] Chen Y., Shi Y., Chen J., Liu H., Pan X., Jin Y., Chen J. (2024). Microwave-assisted synthesis of highly uniform Prussian Blue@Carbon cathode materials for sodium-ion batteries. J. Power Sources.

[98] Ma J., Cui Z., Du Y., Xu Q., Deng Q., Zhu N. (2021). Multifunctional Prussian blue/graphene ink for flexible biosensors and supercapacitors. Electrochim. Acta.

[99] Katic V., dos Santos P.L., dos Santos M.F., Pires B.M., Loureiro H.C., Lima A.P., Queiroz J.C.M., Landers R., Muñoz R.A.A., Bonacin J.A. (2019). 3D Printed Graphene Electrodes Modified with Prussian Blue: Emerging Electrochemical Sensing Platform for Peroxide Detection. ACS Appl. Mater. Interfaces.

[100] Goh G.L., Agarwala S., Yeong W.Y. (2019). Directed and On-Demand Alignment of Carbon Nanotube: A Review toward 3D Printing of Electronics. Adv. Mater. Interfaces.

[101] Fu K., Yao Y., Dai J., Hu L. (2016). Progress in 3D Printing of Carbon Materials for Energy-Related Applications. Adv. Mater..

[102] Zhao B., Sivasankar V.S., Subudhi S.K., Sinha S., Dasgupta A., Das S. (2022). Applications, fluid mechanics, and colloidal science of carbon-nanotube-based 3D printable inks. Nanoscale.

[103] Zhou G., Li M.C., Liu C., Liu C., Li Z., Mei C. (2023). 3D Printed Nitrogen-Doped Thick Carbon Architectures for Supercapacitor: Ink Rheology and Electrochemical Performance. Adv. Sci..

[104] O’ Mahony C., Haq E.U., Silien C., Tofail S.A.M. (2019). Rheological Issues in Carbon-Based Inks for Additive Manufacturing. Micromachines.

[105] Reinhardt K., Hofmann N., Eberstein M. (2018). The importance of shear thinning, thixotropic and viscoelastic properties of thick film pastes to predict effects on printing performance. Proceedings of the EMPC 2017 21st European Microelectronics and Packaging Conference (EMPC) & Exhibition.

[106] Hoath S.D., Hsiao W.-K., Jung S. Properties of PEDOT:PSS from Oscillating Drop Studies. Proceedings of the In NIP & Digital Fabrication Conference.

[107] Dybowska-Sarapuk L., Kielbasinski K., Arazna A., Futera K., Skalski A., Janczak D., Sloma M., Jakubowska M. (2018). Efficient Inkjet Printing of Graphene-Based Elements: Influence of Dispersing Agent on Ink Viscosity. Nanomaterials.

[108] Abodurexiti A., Maimaitiyiming X. (2022). Carbon Nanotubes-Based 3D Printing Ink for Multifunctional “Artificial Epidermis” with Long-Term Environmental Stability. Macromol. Chem. Phys..

[109] Israelachvili J.N. (2011). Intermolecular and Surface Forces.

[110] Yan D., Wang F., Zhao Y., Liu J., Wang J., Zhang L., Park K.C., Endo M. (2009). Production of a high dispersion of silver nanoparticles on surface-functionalized multi-walled carbon nanotubes using an electrostatic technique. Mater. Lett..

[111] Lee J., Hwang D.R., Hong J., Jung D., Shim S.E. (2010). Significance of the Dispersion Stability of Carbon Nanotubes on the Thermal Conductivity of Nylon 610 Nanocomposite. J. Dispers. Sci. Technol..

[112] Ma P.-C., Mo S.-Y., Tang B.-Z., Kim J.-K. (2010). Dispersion, interfacial interaction and re-agglomeration of functionalized carbon nanotubes in epoxy composites. Carbon.

[113] Yadav P., Gupta S.M., Sharma S.K. (2021). A review on stabilization of carbon nanotube nanofluid. J. Therm. Anal. Calorim..

[114] Sun Z., Nicolosi V., Rickard D., Bergin S.D., Aherne D., Coleman J.N. (2008). Quantitative Evaluation of Surfactant-stabilized Single-walled Carbon Nanotubes: Dispersion Quality and Its Correlation with Zeta Potential. J. Phys. Chem. C.

[115] Zhang Y., Chen X., Liu F., Li L., Dai J., Liu T. (2018). Enhanced Coffee-Ring Effect via Substrate Roughness in Evaporation of Colloidal Droplets. Adv. Condens. Matter Phys..

[116] Yan J., Huang S., Lim Y.V., Xu T., Kong D., Li X., Yang H.Y., Wang Y. (2022). Direct-ink writing 3D printed energy storage devices: From material selectivity, design and optimization strategies to diverse applications. Mater. Today.

[117] Saadi M.A.S.R., Maguire A., Pottackal N.T., Thakur M.S.H., Ikram M.M., Hart A.J., Ajayan P.M., Rahman M.M. (2022). Direct Ink Writing: A 3D Printing Technology for Diverse Materials. Adv. Mater..

[118] Khan S., Ali S., Bermak A. (2019). Hybrid Nanomaterials—Flexible Electronics Materials.

[119] Ma T., Li Y., Li A., Niu Y., Cheng H., Yi C., Zhang K. (2025). Nozzle heating with internal channel enhanced aerosol-jet printing with ultrahigh aspect ratio and ultrafine resolution for conformal electronics. Addit. Manuf..

[120] Wang A., Tang X., Zeng Y., Zou L., Bai F., Chen C. (2024). Carbon Fiber-Reinforced PLA Composite for Fused Deposition Modeling 3D Printing. Polymers.

[121] Kudo A., Kanamaru K., Han J., Tang R., Kisu K., Yoshii T., Orimo S.i., Nishihara H., Chen M. (2023). Stereolithography 3D Printed Carbon Microlattices with Hierarchical Porosity for Structural and Functional Applications. Small.

[122] Zhang C.J., McKeon L., Kremer M.P., Park S.-H., Ronan O., Seral-Ascaso A., Barwich S., Coileáin C.Ó., McEvoy N., Nerl H.C. (2023). Additive-free MXene inks and direct printing of micro-supercapacitors. MXenes.

[123] Pandhi T., Chandnani A., Subbaraman H., Estrada D. (2020). A Review of Inkjet Printed Graphene and Carbon Nanotubes Based Gas Sensors. Sensors.

[124] Guo Y., Patanwala H.S., Bognet B., Ma A.W.K. (2017). Inkjet and inkjet-based 3D printing: Connecting fluid properties and printing performance. Rapid Prototyp. J..

[125] Deegan R.D., Bakajin O., Dupont T.F., Huber G., Nagel S.R., Witten T.A. (1997). Capillaryflowasthecause of ring stains fromdried liquid drops. Nature.

[126] Mahajan A., Frisbie C.D., Francis L.F. (2013). Optimization of Aerosol Jet Printing for High-Resolution, High-Aspect Ratio Silver Lines. ACS Appl. Mater. Interfaces.

[127] Seifert T., Sowade E., Roscher F., Wiemer M., Gessner T., Baumann R.R. (2015). Additive Manufacturing Technologies Compared: Morphology of Deposits of Silver Ink Using Inkjet and Aerosol Jet Printing. Ind. Eng. Chem. Res..

[128] Alhendi M., Sivasubramony R.S., Weerawarne D.L., Iannotti J., Borgesen P., Poliks M.D. (2020). Assessing Current-Carrying Capacity of Aerosol Jet Printed Conductors. Adv. Eng. Mater..

[129] Chua C.K., Leong K.F., Lim C.S. (2010). Rapid Prototyping: Principles and Applications.

[130] Maqsood N., Rimašauskas M. (2021). Characterization of carbon fiber reinforced PLA composites manufactured by fused deposition modeling. Compos. Part C Open Access.

[131] Rocha R.G., Ramos D.L.O., de Faria L.V., Germscheidt R.L., dos Santos D.P., Bonacin J.A., Munoz R.A.A., Richter E.M. (2022). Printing parameters affect the electrochemical performance of 3D-printed carbon electrodes obtained by fused deposition modeling. J. Electroanal. Chem..

[132] Zhang W., Liu H., Zhang X., Li X., Zhang G., Cao P. (2021). 3D Printed Micro-Electrochemical Energy Storage Devices: From Design to Integration. Adv. Funct. Mater..

[133] Lewis J.A., Gratson G.M. (2004). Direct writing in three dimensions. Mater. Today.

[134] Lyu Z., Lim G.J.H., Guo R., Kou Z., Wang T., Guan C., Ding J., Chen W., Wang J. (2018). 3D-Printed MOF-Derived Hierarchically Porous Frameworks for Practical High-Energy Density Li–O_2_ Batteries. Adv. Funct. Mater..

[135] Kim N., Park H., Yoon N., Lee J.K. (2018). Zeolite-Templated Mesoporous Silicon Particles for Advanced Lithium-Ion Battery Anodes. ACS Nano.

[136] Golestani E., Javanbakht M., Ghafarian-Zahmatkesh H., Beydaghi H., Ghaemi M. (2018). Tartaric acid assisted carbonization of LiFePO_4_ synthesized through in situ hydrothermal process in aqueous glycerol solution. Electrochim. Acta.

[137] Lawes S., Riese A., Sun Q., Cheng N., Sun X. (2015). Printing nanostructured carbon for energy storage and conversion applications. Carbon.

[138] Wei T.S., Ahn B.Y., Grotto J., Lewis J.A. (2018). 3D Printing of Customized Li-Ion Batteries with Thick Electrodes. Adv. Mater..

[139] Hu J., Jiang Y., Cui S., Duan Y., Liu T., Guo H., Lin L., Lin Y., Zheng J., Amine K. (2016). 3D-Printed Cathodes of LiMn_1−x_Fe_x_PO_4_ Nanocrystals Achieve Both Ultrahigh Rate and High Capacity for Advanced Lithium-Ion Battery. Adv. Energy Mater..

[140] Gao W., Pumera M. (2021). 3D Printed Nanocarbon Frameworks for Li-Ion Battery Cathodes. Adv. Funct. Mater..

[141] Ma J., Zheng S., Chi L., Liu Y., Zhang Y., Wang K., Wu Z.S. (2022). 3D Printing Flexible Sodium-Ion Microbatteries with Ultrahigh Areal Capacity and Robust Rate Capability. Adv. Mater..

[142] Zhang J., Li X.L., Fan S., Huang S., Yan D., Liu L., Valdivia y Alvarado P., Yang H.Y. (2020). 3D-printed functional electrodes towards Zn-Air batteries. Mater. Today Energy.

[143] Wang J., Sun Q., Gao X., Wang C., Li W., Holness F.B., Zheng M., Li R., Price A.D., Sun X. (2018). Toward High Areal Energy and Power Density Electrode for Li-Ion Batteries via Optimized 3D Printing Approach. ACS Appl. Mater. Interfaces.

[144] Zhang T., Ran F. (2021). Design Strategies of 3D Carbon-Based Electrodes for Charge/Ion Transport in Lithium Ion Battery and Sodium Ion Battery. Adv. Funct. Mater..

[145] He Z., Han T., Liu W., Zhou C., Sun J., Zhou J., Li Y.y. (2024). 3D Printed Sodium-Ion Batteries via Ternary Anode Design Affording Hybrid Ion Storage Mechanism. Adv. Energy Mater..

[146] Ji D., Zheng H., Zhang H., Liu W., Ding J. (2022). 3D printed high-performance sodium ion and zinc ion full batteries. J. Alloys Compd..

[147] Katsuyama Y., Kudo A., Kobayashi H., Han J., Chen M., Honma I., Kaner R.B. (2022). A 3D-Printed, Freestanding Carbon Lattice for Sodium Ion Batteries. Small.

[148] Wang Y., Chen C., Xie H., Gao T., Yao Y., Pastel G., Han X., Li Y., Zhao J., Fu K. (2017). 3D-Printed All-Fiber Li-Ion Battery toward Wearable Energy Storage. Adv. Funct. Mater..

[149] Jung J.-W., Nam J.S., Klyukin K., Youn D.-Y., Kim I.-D. (2021). Straightforward strategy toward a shape-deformable carbon-free cathode for flexible Li–air batteries in ambient air. Nano Energy.

[150] Nagy T., Nagy L., Erdélyi Z., Baradács E., Deák G., Zsuga M., Kéki S. (2022). Environmentally friendly high performance Zn-air rechargeable battery using cellulose derivatives: A 3D-printed prototype. J. Energy Storage.

[151] Lin X., Wang J., Gao X., Wang S., Sun Q., Luo J., Zhao C., Zhao Y., Yang X., Wang C. (2020). 3D Printing of Free-Standing “O_2_ Breathable” Air Electrodes for High-Capacity and Long-Life Na–O_2_ Batteries. Chem. Mater..

[152] Li Q., Xu J., Wu X., Zhang T., Li J., Xue Z., Yu M., Luan L., Zhang T., Sun H. (2024). 3D-printed graded graphene aerogel electrode for vanadium redox flow battery. J. Energy Storage.

[153] Zhang X., Zhang D., Liu L., Zhang K., Zhang Y., Zhao J., Han L., Jing M., Liu J., Yan C. (2023). MOF-derived W/Zr bimetallic oxides@Carbon for comprehensively remedying melamine foam electrode defects in vanadium redox flow batteries. Chem. Eng. J..

[154] Li Q., Dong Q., Wang J., Xue Z., Li J., Yu M., Zhang T., Wan Y., Sun H. (2022). Direct ink writing (DIW) of graphene aerogel composite electrode for vanadium redox flow battery. J. Power Sources.

[155] van der Heijden M., Kroese M., Borneman Z., Forner-Cuenca A. (2023). Investigating Mass Transfer Relationships in Stereolithography 3D Printed Electrodes for Redox Flow Batteries. Adv. Mater. Technol..

[156] Rakhi R.B., Ahmed B., Anjum D., Alshareef H.N. (2016). Direct Chemical Synthesis of MnO_2_ Nanowhiskers on Transition-Metal Carbide Surfaces for Supercapacitor Applications. ACS Appl. Mater. Interfaces.

[157] Lethien C., Le Bideau J., Brousse T. (2019). Challenges and prospects of 3D micro-supercapacitors for powering the internet of things. Energy Environ. Sci..

[158] Shen K., Ding J., Yang S. (2018). 3D Printing Quasi-Solid-State Asymmetric Micro-Supercapacitors with Ultrahigh Areal Energy Density. Adv. Energy Mater..

[159] Weng Y., Tan N., Cao Z., Huang B., Lu B., Liu H., You X., Lv J., Guo Y., Tang L. (2025). Tailoring interfacial chemistry and porosity in chitosan-enhanced wood pitch carbon for advanced 3D-printed supercapacitor electrodes. J. Energy Storage.

[160] Idrees M., Ahmed S., Mohammed Z., Korivi N.S., Rangari V. (2020). 3D printed supercapacitor using porous carbon derived from packaging waste. Addit. Manuf..

[161] Khakpour I., Baboukani A.R., Forouzanfar S., Allagui A., Wang C. (2021). In-situ exfoliation and integration of vertically aligned graphene for high-frequency response on-chip microsupercapacitors. J. Power Sources.

[162] Adelowo E., Baboukani A.R., Okpowe O., Khakpour I., Safa M., Chen C., Wang C. (2020). A high-energy aqueous on-chip lithium-ion capacitor based on interdigital 3D carbon microelectrode arrays. J. Power Sources.

[163] Beydaghi H., Abouali S., Thorat S.B., Del Rio Castillo A.E., Bellani S., Lauciello S., Gentiluomo S., Pellegrini V., Bonaccorso F. (2021). 3D printed silicon-few layer graphene anode for advanced Li-ion batteries. RSC Adv..

[164] Delannoy P.E., Riou B., Brousse T., Le Bideau J., Guyomard D., Lestriez B. (2015). Ink-jet printed porous composite LiFePO 4 electrode from aqueous suspension for microbatteries. J. Power Sources.

[165] Sollami Delekta S., Laurila M.-M., Mäntysalo M., Li J. (2020). Drying-Mediated Self-Assembly of Graphene for Inkjet Printing of High-Rate Micro-supercapacitors. Nano-Micro Lett..

[166] Bräuniger Y., Lochmann S., Grothe J., Hantusch M., Kaskel S. (2021). Piezoelectric Inkjet Printing of Nanoporous Carbons for Micro-supercapacitor Devices. ACS Appl. Energy Mater..

[167] Zhou Y., Parker C.B., Joshi P., Naskar A.K., Glass J.T., Cao C. (2020). 4D Printing of Stretchable Supercapacitors via Hybrid Composite Materials. Adv. Mater. Technol..

